# *Alpl* prevents bone ageing sensitivity by specifically regulating senescence and differentiation in mesenchymal stem cells

**DOI:** 10.1038/s41413-018-0029-4

**Published:** 2018-09-11

**Authors:** Wenjia Liu, Liqiang Zhang, Kun Xuan, Chenghu Hu, Shiyu Liu, Li Liao, Bei Li, Fang Jin, Songtao Shi, Yan Jin

**Affiliations:** 1 MS-State Key Laboratory & National Clinical Research Center for Oral Diseases & Shaanxi International Joint Research Center for Oral Diseases, Center for Tissue Engineering, School of Stomatology, Fourth Military Medical University, Xi’an, China; 2 Xi’an Institute of Tissue Engineering and Regenerative Medicine, Xi’an, China; 3 Department of Anatomy and Cell Biology, School of Dental Medicine, University of Pennsylvania, Philadelphia, PA USA

## Abstract

Mutations in the liver/bone/kidney alkaline phosphatase (*Alpl*) gene cause hypophosphatasia (HPP) and early-onset bone dysplasia, suggesting that this gene is a key factor in human bone development. However, how and where *Alpl* acts in bone ageing is largely unknown. Here, we determined that ablation of *Alpl* induces prototypical premature bone ageing characteristics, including bone mass loss and marrow fat gain coupled with elevated expression of p16^INK4A^ (p16) and p53 due to senescence and impaired differentiation in mesenchymal stem cells (MSCs). Mechanistically, *Alpl* deficiency in MSCs enhances ATP release and reduces ATP hydrolysis. Then, the excessive extracellular ATP is, in turn, internalized by MSCs and causes an elevation in the intracellular ATP level, which consequently inactivates the AMPKα pathway and contributes to the cell fate switch of MSCs. Reactivating AMPKα by metformin treatment successfully prevents premature bone ageing in *Alpl*^+/-^ mice by improving the function of endogenous MSCs. These results identify a previously unknown role of *Alpl* in the regulation of ATP-mediated AMPKα alterations that maintain MSC stemness and prevent bone ageing and show that metformin offers a potential therapeutic option.

## Cell biology: diabetes drug prevents bone aging

The diabetes drug metformin restores the ability of stem cells to grow and differentiate into bone-producing osteoblasts, preventing bone aging. The alkaline phosphatase gene (*Alpl*) has been implicated in abnormal bone and tooth development, but its role in bone aging was unclear. The protein it encodes is enriched in the cell membrane of mesenchymal stem cells (MSCs) – precursors of bone-producing osteoblasts – and is involved in the metabolism of ATP, which is implicated in MSC differentiation. Here, Yan Lin at the Xi’an Institute of Tissue Engineering and Regenerative Medicine in China and colleagues demonstrate that Alpl deficiency contributes to bone aging by boosting levels of extracellular ATP. When internalized, this triggers the AMPKα pathway and impairs MSCs’ ability to grow and differentiate. Treatment with metformin reactivates this pathway and prevents premature bone aging.

## Introduction

Alkaline phosphatase (ALP), which was initially identified in 1912,^1^ is a ubiquitous ectoenzyme widely distributed in nature from bacteria to humans.^2^ ALP is a well-known osteoblastic marker and has been widely used as a diagnostic index to evaluate bone formation capacity in osteoporosis.^3,4^ Clinically, a loss-of-function mutation in the liver/bone/kidney ALP (*ALPL)* gene has been linked to a severe skeletal deformity disease termed hypophosphatasia (HPP) that is characterized by bone mass loss and, consequently, pathological fractures.^5–8^ A genetic study investigating *ALPL* in humans and mice strongly suggests that *ALPL* is necessary for postnatal bone formation and that the bone deformities are related to the degree of *ALPL* deficiency.^5–8^ Despite the established function of *ALPL* in bone development, its role in bone ageing remains largely unknown.

Bone ageing, which is the main risk factor for primary osteoporosis, results in a decrease in bone mass and a parallel increase in marrow fat.^2,9–11^ At the cellular level, bone marrow (BM) mesenchymal stem cells (MSCs), which are common progenitors of osteoblasts (OBs) and adipocytes in the BM,^12,13^ undergo senescence along with bone ageing.^9,14,15^ We and other scholars have provided evidence that rescuing the function of MSCs has a significant therapeutic impact on the accrual of the regeneration capacity and bone mass.^16,17^ Tissue nonspecific ALP (TNSALP), which is encoded by *ALPL*, is used as a surface marker for the prospective isolation of MSCs because TNSALP is enriched in the cell membrane.^13,18^ However, whether and how *ALPL* orchestrates the differentiation and senescence of MSCs and subsequently affects bone ageing remain elusive.

Adenosine triphosphate (ATP) has emerged as one of the most versatile extracellular molecules implicated in various cell processes ranging from energy supply to cell-to-cell signaling.^19^ As an ectonucleotidase, TNSALP appears to be involved in the metabolism of nucleotides and can sequentially hydrolyze ATP, ADP and AMP.^20,21^ Thus, the TNSALP level has been reported to be inversely correlated with the extracellular ATP concentration in neurocyte culture medium. MSCs and osteoblastic cell lineages have been shown to spontaneously release ATP,^19,22,23^ which plays a central role in bone physiology.^24,25^ Extracellular ATP is taken up by cells and leads to increased intracellular ATP to supplement the extra energy needs for growth, survival and apoptosis.^26,27^ Therefore, we hypothesize that *Alpl* may regulate ATP homeostasis in MSCs and subsequently lead to the fate switch in MSC differentiation and senescence.

In this study, we reveal that *Alpl* deficiency results in prototypical premature bone ageing characterized by bone mass loss and parallel marrow fat gain coupled with elevated p16^INK4A^ (p16) and p53 expression. Here, our results suggest that bone ageing is partially orchestrated by *Alpl*, which regulates the differentiation and senescence of MSCs. Mechanistically, *Alpl* deficiency in MSCs results in enhanced ATP release and reduced ATP hydrolysis, which is, in turn, internalized by MSCs and consequently contributes to the cell fate change by regulating the AMPKα pathway. These data have far-reaching implications for the understanding of the role of *Alpl* in bone ageing and the development of a new treatment method via the revaluation of *Alpl*.

## Results

### Decrease in *Alpl* in the BM leads to bone ageing

**Fig. 1:**
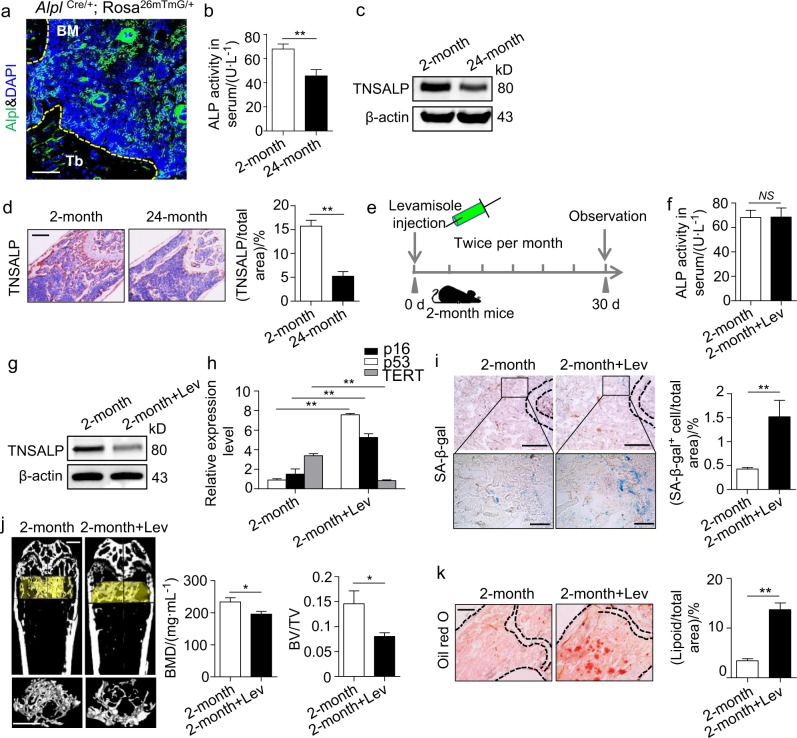
Declined expression of *Alpl* in the bone marrow is associated with bone ageing characteristics. **a** Representative image of immunostaining analysis showing *Alpl* (green) in the BM of 2-month-old *Alpl* ^Cre/+^; Rosa^26mTmG/+^ mice. Scale bar: 50 μm. **b,** **c** ALP activities in serum and expression levels in the BM of young (2-month) and old (24-month) mice were analyzed by an ALP activity assay and a western blotting analysis. **d** Immunohistochemical analysis of TNSALP (brown) of the femoral diaphysis. Quantification of the TNSALP^+^ area is indicated on the right. Scale bars, 500 μm. **e** Scheme of the levamisole injection mouse model. We injected 10 mg⋅kg^–1^ levamisole into the femoral bone marrow cavity of 2-month-old mice every 2 weeks for 1 month (total of two injections), and NaCl was used as a control. **f**, **g** ALP activities and expression levels were analyzed by an ALP activity assay and a western blotting analysis. **h** Expression levels of the ageing-specific genes *p16, p53* and *TERT* were examined via qRT-PCR. **i** SA-β-gal staining in the BM and quantification of β-gal^+^ (blue) area are indicated on the right. Scale bars, 500 μm (upper). Scale bars, 100 μm (lower). **j** μCT images and quantification of bone mineral density (BMD) and bone volume/total volume (BV/TV) of the femur. Scale bars, 1 mm. **k** Oil Red O staining images and quantitative analyses of the area of adipose tissue over the total area of the femoral diaphysis. Scale bars, 500 μm. Young group, *n* = 6, Old group, *n* = 6; all injection groups, *n* = 8. The data are presented as the means ± standard deviation (s.d.) of each experiment performed in triplicate. *P *<* 0.05, **P *<* 0.01, *NS* not significant. Unpaired two-tailed Student’s *t*-test

Bone ageing is often manifested as a progressive decrease in bone mass and a parallel increase in marrow fat as shown by a microcomputer tomography (μCT) analysis and Oil Red O staining (Supplementary Fig. 1a, b). *Alpl* is expressed in many organs and tissues, especially in the kidney, liver and bone, as shown in *Alpl*^Cre/+^; Rosa^26mTmG/+^ mice (Supplementary Fig. 2a). Notably, more *Alpl*^+^ cells were observed in the BM than in the trabecular bone (Fig. 1a). Ageing-related *p16* and *p53* expression was elevated from adolescence (2 months) to advanced age (24 months) (Supplementary Fig. 1c). However, both ALP activity and TNSALP expression gradually declined with ageing (Fig. 1b–d) as indicated by the ALP activity assay, western blotting and immunohistochemical analyses. The inverse correlation was also recapitulated in a senescence-accelerated mouse (SAM) R1/P6 model (Supplementary Fig. 1d-h). Thus, using two ageing mouse models, we revealed that bone ageing characteristics could be associated with a decreased *Alpl* expression in the BM.

To further investigate the role of *Alpl* in bone ageing, we injected 10 mg⋅kg^–1^ levamisole, which is an ALP activity inhibitor, into the femoral BM cavity of 2-month-old mice every 2 weeks for 1 month (total of two injections), and NaCl was used as a control (Fig. 1e). The serum ALP activity was unchanged, but TNSALP expression in the BM progressively decreased 1 month after the injection (Fig. 1f, g). Notably, the inhibition of *Alpl* expression induced more senescence-associated β-galactosidase activity (SA-β-gal)^+^ cells in the BM and higher expression levels of ageing-related *p16* and *p53* (Fig. 1h, i). The μCT analysis and Oil Red O staining showed that the bone mass was decreased, whereas the marrow adiposity was significantly increased compared to that in the non-injection group (Fig. 1j, k). These observations indicate that *Alpl* expression in the BM is inversely correlated with the progression of bone ageing.

### Ablation of *Alpl* results in premature bone ageing characteristics along with senescence and impaired differentiation of MSCs

**Fig. 2:**
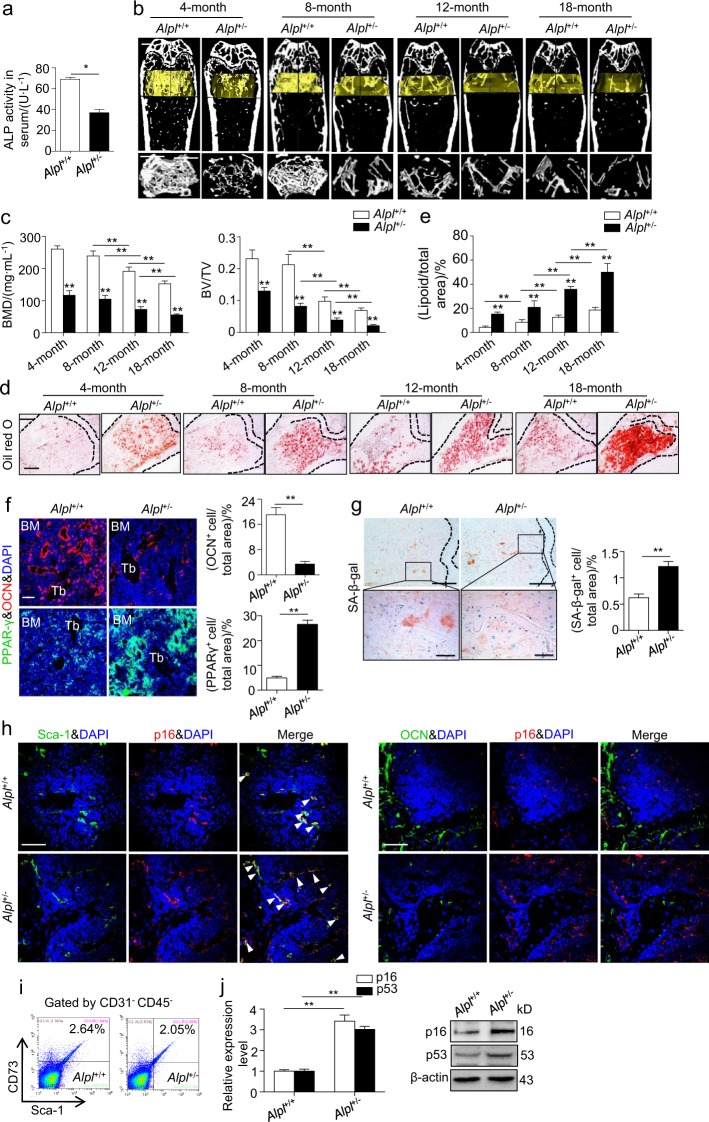
Heterozygous *Alpl*^+/-^ mice exhibit age-related bone mass loss and marrow fat gain, mimicking premature bone ageing. **a** Serum ALP activities in 4-month-old *Alpl*^+/+^ and *Alpl*^+/-^ mice were analyzed by an ALP activity assay. **b,** **c** μCT images and quantification of BMD and BV/TV. Scale bars, 1 mm. **d,** **e** Oil Red O staining images and quantitative analysis of the area of adipose tissue over the total area of the proximal femoral diaphysis. Scale bars, 500 μm. **f** Immunostaining analysis of OCN (red), PPAR-γ (green) and nuclear staining (blue, DAPI) of the proximal femoral diaphysis. Quantification of OCN^+^ and PPAR-γ^+^ cells is indicated in the right panel. Scale bars, 200 μm. **g** SA-β-gal staining in the BM and quantification of the β-gal^+^ area are indicated on the right. Scale bars, 500 μm (upper). Scale bars, 100 μm (lower). **h** Immunostaining showed overlap between p16 (red) and Sca-1/OCN (green) in the *Alpl*^+/+^ and *Alpl*^+/-^ BM. The white arrows indicate the p16^+^Sca-1^+^ cells. Scale bar: 100 μm. **i** Representative dot plot of MSC FASC analysis of the expression (+) or absence (-) of the following standard markers: CD73^+^Sca1^+^CD45^-^CD31^-^. **j** Expression levels of the ageing-specific genes *p16* and *p53* in FASC sorted cells were examined via qRT-PCR and western blotting. *n* = 6 per group. The data are presented as the means ± s.d. of each experiment performed in triplicate. *P *<* 0.05, **P < 0.01, unpaired two-tailed Student’s *t*-test

To further investigate the regulatory role of *Alpl* in bone ageing, we used *Alpl*-knockout (C57BL/6J*-Alpl*^+/-^) mice. In most cases, homozygous *Alpl*^*-/-*^ mice die at 20 days of age.^28^ Consequently, these mice are not appropriate for studying bone phenotype changes with age. In contrast, heterozygous *Alpl*^+/-^ mice survive normally and show no changes in size, weight or appearance compared with wild-type (WT) littermates (*Alpl*^+/+^) (Supplementary Fig. 2b, c). A nearly 50% reduction in serum ALP activity was observed in 4-month-old *Alpl*^+/-^ mice relative to that in the WT group (Fig. 2a). The μCT analysis revealed that the *Alpl*^+/-^ mice exhibited age-related bone loss compared with the WT controls at 4, 8, 12 and 18 months as shown by a decreased BM density (BMD) and bone volume/total volume (BV/TV) (Fig. 2b, c). In contrast, compared with the WT controls, the BM adipose tissue gradually increased in the femur of the *Alpl*^+/-^ mice as the animals aged as evidenced by Oil Red O staining (Fig. 2d, e). Moreover, fewer osteocalcin (OCN)^+^ cells but more peroxisome proliferator-activated receptor γ (PPARγ) ^+^ cells were observed in the BM of the *Alpl*^+/-^ mice (Fig. 2f), which is consistent with the perturbations in bone and adipose tissue in the *Alpl*^+/-^ mice.

In addition to the age-related bone loss and marrow fat gain in the *Alpl*^+/-^ mice, the expression of *p16* was higher, whereas the expression of *TERT* declined in most organs of the 4-month-old *Alpl*^+/-^ mice, especially in tissues enriched in *Alpl* (Supplementary Fig. 2d, e). Specifically, the number of SA-β-gal^+^ cells and the expression of *p16* were obviously increased in the *Alpl*^+/-^ BM, but *TERT* expression was decreased relative to that in the WT controls (Fig. 2g and Supplementary Fig. 2d, e). Although the *Alpl*^+/-^ mice displayed age-related gradual bone loss, osteoclastogenesis in the *Alpl*^+/-^ mice was almost the same as that in their WT controls as evidenced by the μCT analysis of cortical bone, tartrate-resistant acidic phosphatase (TRAP) staining and enzyme-linked immunosorbent assay (ELISA) analysis of carboxy-terminal telopeptide of type 1 collagen (CTX-1) (Supplementary Fig. 2g-i), indicating that *Alpl*-deficient bone displays premature ageing-related characteristics independent of osteoclastogenesis.

MSCs represent a promising cell source for the maintenance of bone homeostasis^29,30^ and undergo senescence with bone ageing.^9,14,15^ Interestingly, we observed that the p16^+^ senescent cells were mainly colocalized with Sca-1^+^ cells but barely colocalized with OCN^+^ cells in both the WT and *Alpl*^+/-^ BM (Fig. 2h), indicating that the bone ageing phenotype of the *Alpl*^+/-^ mice may be related to the senescence of MSCs rather than OBs. To further validate this result, we tested the expression of *p16* and *p53* in the WT and *Alpl*^+/-^ BM sorted by flow cytometry as CD73^+^Sca1^+^CD31^–^CD45^–^. The increased *p16* and *p53* expression levels in the sorted MSCs were consistent with the results observed in the BM (Fig. 2i, j and Supplementary Fig. 2d, e). Taken together, these results suggest that the premature bone ageing characteristics caused by *Alpl* deficiency are probably due to the senescence and impaired differentiation of MSCs.

### *Alpl* governs the osteo-adipogenic balance of MSCs and prevents cell senescence

**Fig. 3:**
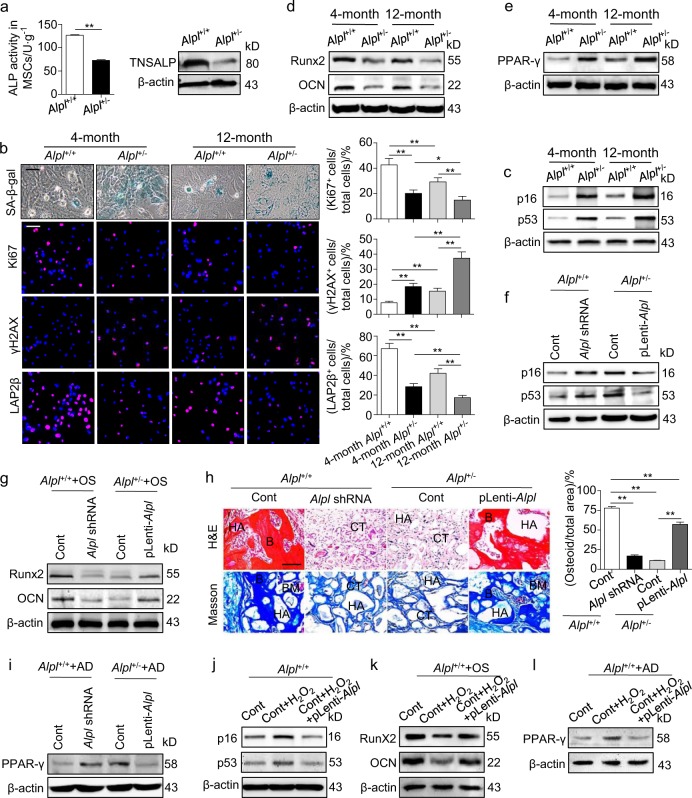
*Alpl* controls the osteo-adipogenic balance in MSCs and prevents their senescence. **a** ALP activities and expression levels in *Alpl*^+/+^ and *Alpl*^+/-^ MSCs were examined by an ALP activity assay and a western blotting analysis. **b** SA-β-gal staining and Ki67, γH2AX and LAP2β immunostaining of first-passage MSCs from *Alpl*^+/+^ and *Alpl*^+/-^ mice at 4 and 12 months. Quantification of Ki67^+^, γH2AX^+^ and LAP2β^+^ is shown in the right panel. Scale bars: 50 μm. **c** Expression levels of p16 and p53 in MSCs from *Alpl*^+/+^ and *Alpl*^+/-^ mice at 4 and 12 months were examined by a western blotting analysis. **d,** **e** Expression levels of Runx2, OCN and PPAR-γ in MSCs from *Alpl*^+/+^ and *Alpl*^+/-^ mice at 4 and 12 months were examined by a western blotting analysis on day 7 after the osteogenic/adipogenic induction. **f** Downregulated *Alpl* expression in *Alpl*^+/+^ MSCs and upregulated expression in *Alpl*^+/-^ MSCs by lentiviral vectors. Expression levels of the ageing-specific genes p16 and p53 in MSCs were examined by a western blotting analysis. **g** Expression levels of Runx2 and OCN were examined by a western blotting analysis on day 7 after the osteogenic induction. **h** HE/Masson’s trichrome staining and quantitative analysis revealed the formation of bone (B), bone marrow (BM) and collagen fiber (CF) around the HA/TCP (HA) carrier after the MSCs were implanted into nude mice. Scale bars, 200 μm. **i** PPAR-γ expression was examined on day 7 after the adipogenic induction by western blotting. **j** *Alpl*^+/+^ MSCs were treated with 50 μM H_2_O_2_ for 24 h with or without the overexpression of *Alpl*; then, the medium was replaced with normal medium, followed by incubation for another 24 h. Expression levels of p16 and p53 were examined by a western blotting analysis. **k,** **l** *Alpl*^+/+^ MSCs were treated with 50 μM H_2_O_2_ for 24 h with or without *Alpl* overexpression, and then, the medium was changed to induction medium, followed by incubation for 7 d. Expression levels of Runx2, OCN and PPARγ were examined by a western blotting analysis. *n* = 6 per groups. The data are shown as the means ± s.d. of each independent experiment performed in triplicate. *P *<* 0.05, **P < 0.01. **a** Unpaired two-tailed Student’s *t-*test. **b**, **h** One-way analysis of variance (ANOVA)

To determine the role of *Alpl* in MSC senescence and differentiation, we first compared the function of cultured MSCs between 4- and 12-month-old WT and *Alpl*^+/-^ mice. The MSCs were flushed from the BM and cultured in vitro (Supplementary Table 1). ALP activity and TNSALP expression were both decreased by more than half in the *Alpl*^+/**-**^ MSCs compared with those in the WT MSCs (Fig. 3a). The *Alpl*^+/-^ MSCs had relatively fewer Ki67^+^ cells and ageing-associated marker LAP2β^+^ cells^31^ than the WT controls in the 4- and 12-month-old mice. However, many γ-H2AX^+^ cells were observed, indicating that the DNA damage response was elevated, and SA-β-gal^+^ cells and gradually increasing *p16* and *p53* expression were observed in the MSCs from the 4- and 12-month-old *Alpl*^+/-^ mice (Fig. 3b, c). In addition, the *Alpl*^+/-^ MSCs manifested age-related enhanced adipogenic differentiation at the expense of osteogenic differentiation as shown by the decreased Runx2 and OCN levels and increased PPARγ expression (Fig. 3d, e). These results indicate that *Alpl* deficiency in MSCs results in age-related senescence and impaired lineage differentiation.

To further validate the regulatory role of *Alpl* in the fate switch of MSCs, we up- or downregulated the *Alpl* level in cultured MSCs via lentiviral transduction (Supplementary Fig. 3). The downregulation of *Alpl* in the WT MSCs significantly induced MSC senescence, whereas the recovery of *Alpl* expression in the *Alpl*^+/-^ MSCs prevented cell senescence as evidenced by the expression of *p16* and *p53* (Fig. 3f). In addition, the inhibition of *Alpl* in the WT MSCs impaired their osteogenic differentiation but enhanced adipogenic differentiation, mimicking the *Alpl*^+/**-**^ MSC phenotype. In contrast, the recovery of *Alpl* expression in the *Alpl*^+/-^ MSCs rescued their impaired osteogenic differentiation at the expense of adipogenic differentiation as confirmed by the Runx2, OCN and PPARγ expression in vitro and the regeneration of new bone in vivo (Fig. 3g–i). More convincingly, the increased expression of *Alpl* in the WT MSCs partially prevented the senescence caused by a high concentration of H_2_O_2_ and recovered their osteo-adipogenic differentiation (Fig. 3j–l). Therefore, *Alpl* is necessary for lineage alteration in MSCs and prevents their senescence, subsequently affecting the phenotype of postnatal bone.

### *Alpl* deficiency induces the release of ATP, which is, in turn, internalized by MSCs and causes their dysfunction

Given that TNSALP has been reported to hydrolyze inorganic pyrophosphate (PPi) to promote OB mineralization,^32^ we investigated whether TNSALP regulates the differentiation of MSCs through PPi. The extracellular PPi concentrations were indeed varied according to the *Alpl* level, but the intracellular PPi levels were unaffected. Additionally, exogenous PPi merely inhibited the osteogenic differentiation of MSCs but barely affected their adipogenic differentiation (Supplementary Fig. 4), suggesting that PPi is not a major factor in the MSC lineage alteration caused by *Alpl*.

**Fig. 4:**
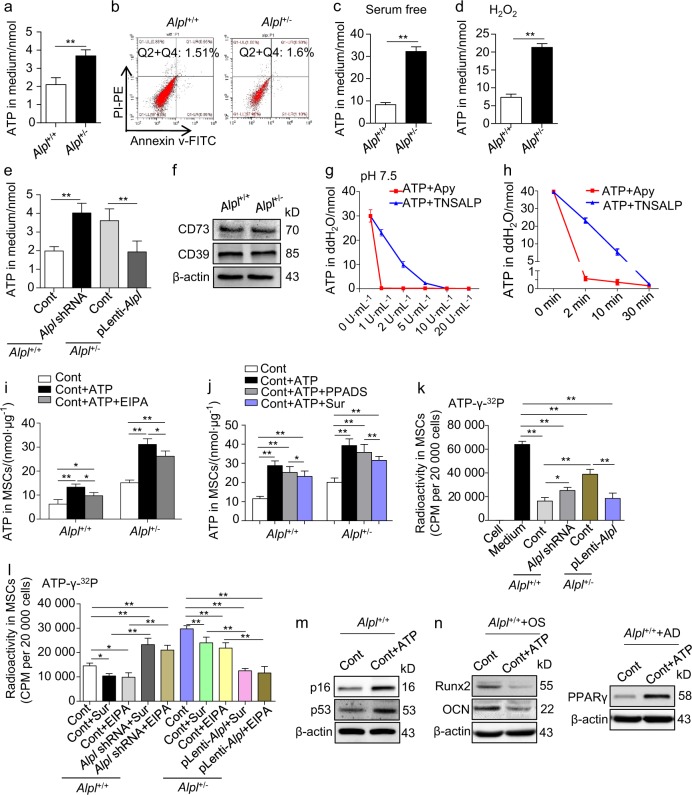
*Alpl* deficiency induces an elevation in extracellular ATP, which is internalized by MSCs and causes their dysfunction. **a** Extracellular ATP concentrations in *Alpl*^+/+^ and *Alpl*^+/-^ MSC medium were assayed by a regular ATP concentration assay. **b** Apoptosis of *Alpl*^+/+^ and *Alpl*^+/-^ MSCs was analyzed by flow cytometry. **c**, **d** Extracellular ATP concentrations were assayed 1 h after FBS deprivation and H_2_O_2_ induction (50 mmol⋅L^–1^). **e** Extracellular ATP concentrations were assayed 48 h after transduction with different lentiviral vectors. **f** Expression levels of CD73 and CD39 in *Alpl*^+/+^ and *Alpl*^+/-^ MSCs were analyzed by western blotting. **g** ATP concentrations were assayed after 2 min of treatment with 0, 1, 2, 5, 10 or 20 U⋅mL^–1^ of TNSALP and 2 U⋅mL^–1^ of ATP-apyrase in the presence of 20 nmol⋅L^–1^ ATP in double-distilled water (dd H_2_O, pH 7.5). **h** ATP concentrations were assayed at 0 min, 2 min, 10 min and 30 min after treatment with 10 U⋅mL^–1^ of TNSALP and 2 U⋅mL^–1^ of ATP-apyrase in the presence of 20 nmol⋅L^–1^ ATP in dd H_2_O. **i**, **j** *Alpl*^+/+^ and *Alpl*^+/-^ MSCs were treated with 10 μmol⋅L^–1^ ATP in the presence or absence of 50 μmol⋅L^–1^ ethyl isopropyl amiloride (EIPA), 30 μmol⋅L^–1^ pyridoxal phosphate-6-azo (PPADS) or 100 μmol⋅L^–1^ suramin (Sur), and the intracellular ATP concentrations were assayed 1 h after treatment. **k** Intracellular radioactivity was examined after a 1-h treatment with ATP-γ-^32^P in the different lentivirus transduction groups. **l** Intracellular radioactivity was examined after a 1-h treatment with ATP-γ-^32^P in different lentivirus transduction groups treated with 100 μmol⋅L^–1^ suramin and 50 μmol⋅L^–1^ EIPA. **m,** **n** *Alpl*^+/+^ MSCs were treated with 10 μmol⋅L^–1^ ATP, and the ageing-specific genes were analyzed after 48 h. Expression levels of Runx2, OCN and PPARγ were examined by western blotting on day 7 after induction. *n* = 6 per group. The data are presented as the means ± s.d. of each independent experiment performed in triplicate. *P *<* 0.05, **P < 0.01. **a**-**d**, **g**-**h**, **i**-**j** Unpaired two-tailed Student’s *t-*test. **e**, **k**, **l** One-way analysis of variance (ANOVA)

TNSALP is also an efficient ATP ectonucleotidase,^33,34^ and extracellular ATP has been reported to regulate the proliferation, migration and differentiation of MSCs.^35^ Therefore, we tested whether ATP was involved in the *Alpl*-mediated cell fate choice of MSCs. As predicted, the extracellular ATP level in the *Alpl*^+/-^ MSCs was significantly higher than that in the WT MSCs (Fig. 4a). MSCs have been shown to spontaneously release ATP,^19,22^ and the major release mechanism is through tissue and cell damage.^19,25^ However, we observed that apoptosis in the *Alpl*^+/-^ MSCs was identical to that in the WT MSCs (Fig. 4b), indicating that the higher extracellular ATP level in the *Alpl-*deficient MSCs may not be caused by cell death. Therefore, we hypothesized that *Alpl* may regulate ATP release. To confirm this hypothesis, we induced MSCs by serum starvation and H_2_O_2_ treatment. After the induction, the *Alpl*^+/-^ MSCs, but not the WT MSCs, released a large amount of ATP (Fig. 4c, d). More strikingly, the downregulation of *Alpl* led to increased extracellular ATP in the WT MSCs, whereas enforcing expression in the *Alpl*^+/-^ MSCs significantly reduced the extracellular ATP level (Fig. 4e), suggesting that *Alpl* in MSCs probably regulates ATP release.

Nucleoside triphosphate dephosphorylase (CD39) and ecto-5’-nucleotidase (CD73) are also classical cell surface ATP apyrases that hydrolyze ATP/ADP to AMP and adenosine, respectively.^27,36^ However, in contrast to the expression of TNSALP, the expression of CD39 and CD73 did not significantly differ between the WT and *Alpl*^+/-^ MSCs (Fig. 4f). Additionally, in contrast to the classical ATP-apyrase, the enzymatic reaction experiments revealed that the hydrolysis of TNSALP occurred in a dose- and time-dependent manner (Fig. 4g, h). Due to the decrease in TNSALP activity and expression by nearly 50% in the *Alpl*^+/-^ MSCs (Fig. 3a), their ATP hydrolytic ability was probably weaker than that of the WT MSCs, further resulting in a higher extracellular ATP level. Therefore, coupled with more ATP release from *Alpl*^+/-^ MSCs, the extracellular ATP level was further increased due to the *Alpl* deficiency.

We further noticed that similar to the extracellular ATP level, the intracellular ATP concentrations were changed according to the *Alpl* level (Supplementary Fig. 5a-c). Additionally, this elevation due to the *Alpl* deficiency was independent of ATP synthesis (Supplementary Fig. 5d). Thus, we investigated how extracellular ATP affects the intracellular ATP level and, subsequently, cell function. The intracellular ATP concentration in the cells treated with ATP and ethyl isopropyl amiloride (EIPA), which is a macropinocytosis inhibitor,^37,38^ was only reduced by nearly 16%–20% compared with that in the group that was only treated with ATP but was still 70% higher than the baseline level in the control group (Fig. 4i), indicating that macropinocytosis is not a major determinant of the intracellular ATP increase. Additionally, previous studies have reported that extracellular ATP regulates cell function by activating ionotropic P2X and metabotropic P2Y receptors.^39–41^ Thus, we blocked these receptors with pyridoxal phosphate-6-azo (PPADS) and suramin, which are nonselective P2X and P2Y receptor antagonists, respectively. Similar to the EIPA experiment, the ATP levels were decreased by no >20% in both the WT and *Alpl*^+/-^ MSCs treated with PPADs and suramin (Fig. 4j), indicating that the intracellular ATP levels in MSCs are not mainly affected by traditional purinergic signaling.

To further elucidate how the intracellular ATP levels were affected by the extracellular ATP levels, we performed radioisotope experiments. The results showed that the intracellular radioactivity of ATP-γ-^32^P was 2.5−fold higher in the *Alpl*^+/-^ MSCs than that in the WT MSCs after adding radioactive ATP to the cell culture medium for 1 h. Furthermore, the intracellular radioactive profile was inversely correlated with the *Alpl* level (Fig. 4k and Supplementary Fig. 5e). More importantly, the radioactivity could not be blocked by either the EIPA or suramin treatment, which is consistent with the regular ATP assays (Fig. 4l). As predicted, exogenous ATP added to the WT MSC culture medium could cause increased intracellular ATP that not only induced senescence but also inhibited osteogenesis, promoting adipogenesis (Fig. 4m, n). Collectively, these findings suggest that *Alpl* deficiency induces the excessive elevation of extracellular ATP, which is subsequently internalized by MSCs mainly by directly entering the cytoplasm and causing their fate changes.

### ATP induces MSC dysfunction by repressing the AMPKα pathway

**Fig. 5:**
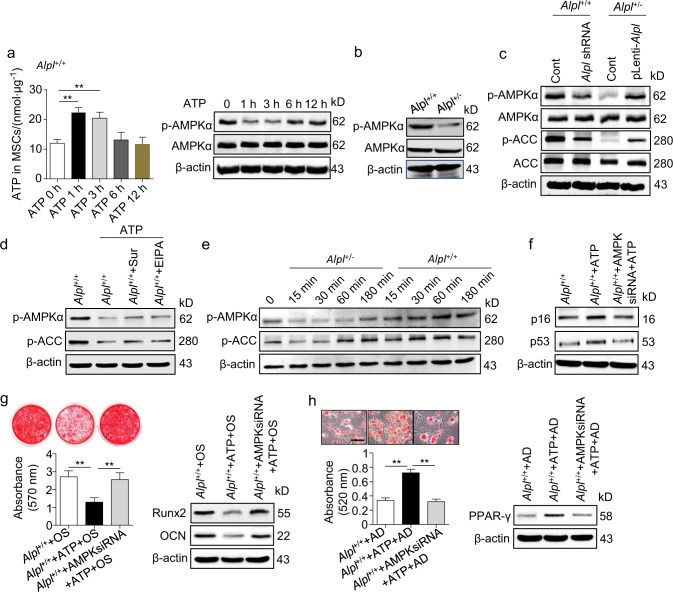
ATP-mediated AMPKα pathway inactivation contributes to MSC dysfunction. **a** *Alpl*^+/+^ MSCs were treated with 10 μmol⋅L^–1^ ATP. Intracellular ATP concentrations and expression levels of AMPKα and p-AMPKα were examined 0, 1, 3, 6 and 12 h after treatment. **b** Expression levels of AMPKα and p-AMPKα in the *Alpl*^+/+^ and *Alpl*^+/-^ MSCs were examined by western blotting. **c** Expression levels of AMPKα, p-AMPKα, ACC and p-ACC in *Alpl*^+/+^ and *Alpl*^+/-^ MSCs transfected with lentivirus were analyzed by western blotting. **d** *Alpl*^+/+^ MSCs were treated with 10 μmol⋅L^–1^ ATP with or without 50 μmol⋅L^–1^ EIPA and 100 μmol⋅L^–1^ suramin, and the expression levels of p-AMPKα and p-ACC were analyzed by western blotting. **e** 293T cells were treated with medium of *Alpl*^+/+^ and *Alpl*^+/-^ MSCs, and the expression levels of p-AMPKα and p-ACC were analyzed by western blotting. **f-h** Downregulated AMKPα expression in *Alpl*^+/+^ MSCs and treatment with or without 10 μmol⋅L^–1^ ATP. Ageing-specific genes were analyzed at 48 h by western blotting. Alizarin Red and Oil Red O staining and quantifications were performed on day 21 and day 14 after the osteogenic/adipogenic induction (OS/AD). Expression levels of Runx2, OCN and PPAR-γ were examined by western blotting on day 7 after induction. Scale bars, 100 μm. *n* = 6 per group. The data are presented as the means ± s.d. of each independent experiment performed in triplicate. **P < 0.01. One-way analysis of variance (ANOVA)

Subsequently, we aimed to obtain insight into how ATP regulates the function of MSCs. The AMP-activated protein kinase α (AMPKα) pathway is directly regulated by the ATP level.^42^ We observed that the addition of ATP to the culture medium of WT MSCs caused an increase in intracellular ATP, thereby inhibiting the phosphorylation of AMPKα-Thr172 (p-AMPKα), which peaked 1 and 3 h after incubation (Fig. 5a). Thus, we investigated the relationship between the AMPKα pathway and *Alpl* level. As expected, p-AMPKα, but not total AMPKα, was significantly decreased in the *Alpl*^+/-^ MSCs (Fig. 5b). More convincingly, the phosphorylation of AMPKα and its downstream target acetyl-CoA carboxylase (ACC) were correlated with the level of *Alpl*, whereas the total protein did not change as indicated by a western blotting analysis (Fig. 5c). Importantly, this ATP-mediated inactivation of the AMPKα pathway was not rescued by the suramin or EIPA treatment (Fig. 5d). To further validate our observations, we collected the culture medium of WT and *Alpl*^+/-^ MSCs. The supernatant of the *Alpl*^+/-^ MSCs suppressed the expression levels of p-AMPKα and p-ACC in 293T cell lines (Fig. 5e), indicating that the higher extracellular ATP levels due to the *Alpl* deficiency could inactivate the AMPKα pathway.

The AMPKα pathway also plays a role in bone physiology. The activation of AMPK promotes bone formation in vitro, and the deletion of the α or β subunit of AMPK decreases bone mass in mice.^43,44^ We also observed that the inhibition of the AMPKα pathway in MSCs promoted adipogenic differentiation at the expense of osteogenic differentiation (Supplementary Fig. 6). More strikingly, the addition of ATP could not induce senescence and impair the lineage differentiation of WT MSCs after the knockdown of AMPKα by small interfering RNA (siRNA) (Fig. 5f–h). Taken together, these data demonstrate that ATP directly represses the AMPKα pathway, thereby contributing to the alterations in the differentiation and senescence of MSCs.

### Metformin rescues endogenous MSC function and prevents premature bone ageing in *Alpl*^+/-^ mice

As *Alpl* and the AMPKα pathway both effectively regulate the function of MSCs, we compared the therapeutic effect of *Alpl* overexpression and AMPKα activation on the recovery of *Alpl*^*+/-*^ MSCs. Metformin is a common activator of the AMPKα pathway^3^ and has been recently used as an anti-ageing drug.^45^ Notably, metformin exhibited a stronger capacity to rescue the differentiation of *Alpl*^+/-^ MSCs than *Alpl* expression as evidenced by the increased osteogenic differentiation and decreased adipogenic differentiation (Supplementary Fig. 7a, b). In addition, compared with the *Alpl*^+/-^ mice in the non-treated groups, the skeletal deformities in the *Alpl*^+/-^ mice given metformin-treated *Alpl*^+/-^ MSCs were ameliorated (Supplementary Fig. 7c, d).

**Fig. 6:**
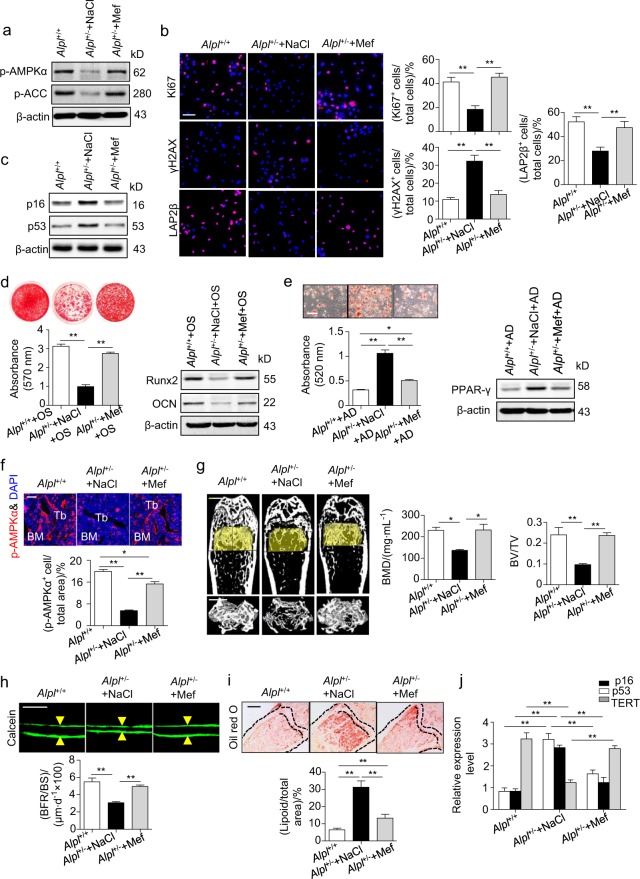
Metformin treatment prevents bone ageing in *Alpl*^+/-^ mice by rescuing the impaired function of MSCs. We injected 60 mg⋅kg^–1^ metformin into the femoral bone marrow cavity of 4-month-old *Alpl* ^+/-^ mice every 2 weeks for 1 month (total of two injections), and NaCl was used as a control. **a** Expression levels of p-AMPKα and p-ACC in MSCs from three groups were analyzed by western blotting. **b** Immunostaining of Ki67, γH2AX and LAP2β in MSCs from control and metformin-treated mice. Quantification of Ki67^+^, γH2AX^+^ and LAP2β^+^ is shown in the right panel. Scale bars: 50 μm. **c** Expression levels of p16 and p53 in MSCs were examined by western blotting. **d** Alizarin Red staining and quantification of mineralized nodules were performed on day 21 after the osteogenic induction (OS) in the MSCs from *Alpl*^+/+^ and *Alpl* ^+/-^ mice injected with NaCl or metformin. Expression levels of Runx2 and OCN were examined by western blotting on day 7 after induction. **e** Oil Red O staining and quantification of fat depots were performed on day 14 after the adipogenic induction (AD). Scale bars, 100 μm. PPAR-γ expression was examined on day 7 after induction by western blotting. **f** Immunostaining analysis showing the expression of p-AMPKα (red) and nuclear staining (blue, DAPI) in the proximal femoral diaphysis. Quantification of p-AMPKα^+^ cells is indicated in the bottom panel. **g** μCT images and quantification of BMD and BV/TV. Scale bars, 1 mm. **h** Images of calcein double labeling of trabecular bone with quantification of BFR/BS. Scale bars, 50 μm. **i** Oil Red O staining images and quantitative analysis of the area of adipose tissue over the total area of the proximal femoral diaphysis. Scale bars, 500 μm. **j** Expression levels of ageing-specific genes were examined via qRT-PCR. *n* = 8 per group. The data are presented as the means ± s.d. of each independent experiment performed in triplicate. *P *<* 0.05, **P < 0.01. One-way analysis of variance (ANOVA)

To determine whether metformin exerted a therapeutic effect in vivo, we injected 60 mg kg^–1^ metformin into the femoral BM cavity of 4-month-old *Alpl*^+/-^ mice twice per month for 1 month, and 0.9% NaCl was used as the vehicle and control. The expression levels of p-AMPKα and p-ACC were elevated in the *Alpl*^+/-^ MSCs after the metformin injections (Fig. 6a). Consequently, more Ki67^+^ cells and LAP2β^+^ cells but fewer γH2AX^+^ cells were observed in the injection group compared with those in the non-treatment group (Fig. 6b). Moreover, a declined *p16* and *p53* expression was also detected in the metformin-treated group (Fig. 6c), indicating that the senescence characteristics of the endogenous *Alpl*^+/-^ MSCs were rescued by the metformin treatment. Along with the senescence recovery in the *Alpl*^+/-^ MSCs, lineage differentiation was rescued by metformin as evidenced by an enrichment in mineralized nodules, increased expression levels of Runx2 and OCN, and reduced fat depots and PPARγ expression (Fig. 6d, e). Importantly, we observed that metformin restored p-AMPKα expression in the BM of the *Alpl*^+/-^ mice (Fig. 6f). Thus, the premature bone ageing in the *Alpl*^+/-^ mice was attenuated, representing a 1.9-fold BMD, 1.9-fold bone formation rate/bone surface (BFR/BS) and 2.5-fold BV/TV improvement compared with those in the NaCl group (Fig. 6g, h). In contrast, both the adipose tissue and expression of *p16* and *p53* in the BM of the *Alpl*^+/-^ mice were reduced by >50% after the metformin treatment (Fig. 6i, j). Notably, the therapeutic effect of metformin on rescuing the premature bone ageing characteristics was also validated in 12-month-old *Alpl*^+/-^ mice as evidenced by an increased bone formation capacity and bone mass and decreased adipogenesis and ageing-related gene expression (Supplementary Fig. 8). Taken together, reactivating the AMPKα pathway by local metformin administration rescues premature bone ageing in *Alpl*^+/-^ mice probably by restoring the function of endogenous MSCs.

### *Alpl*-controlled cell fate is also observed in human MSCs

**Fig. 7:**
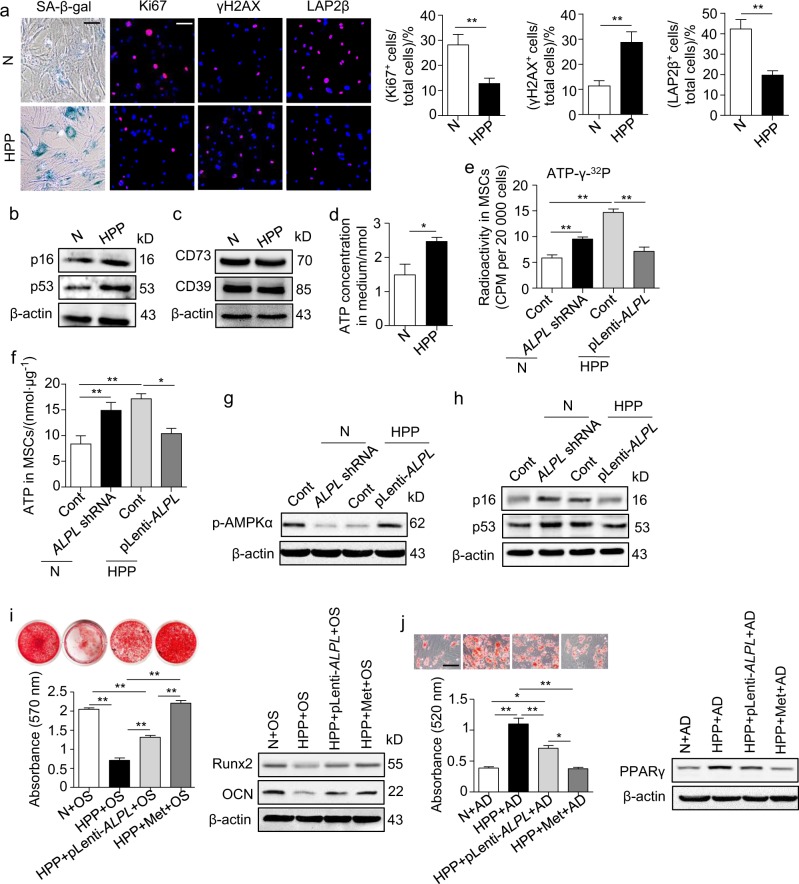
*Alpl* also controls the differentiation and senescence of human MSCs via ATP-mediated inactivation of the AMPKα pathway. **a** SA-β-gal staining and Ki67, γH2AX and LAP2β immunostaining of third-passage MSCs from normal controls and HPP patients. Quantification of Ki67^+^, γH2AX^+^ and LAP2β^+^ is indicated in the right panel. Scale bars: 50 μm. **b** Expression levels of ageing-specific genes in normal and HPP MSCs were examined by western blotting. Scale bars, 50 μm. **c** Expression levels of CD73 and CD39 in normal and HPP MSCs were examined by western blotting. **d** Extracellular ATP concentrations in normal and HPP MSC medium were examined by a regular ATP concentration assay. **e** Intracellular radioactivity was examined after a 1-h treatment with ATP-γ-^32^P in different lentiviral vector transduction groups. **f** Intracellular ATP concentrations were assayed 48 h after the transduction of different lentiviral vectors. **g** Western blotting analysis of p-AMPKα expression in normal control and the *ALPL* shRNA, HPP control and pLenti-*ALPL* groups. **h** Expression levels of p16 and p53 were assayed 48 h after the transduction of different lentiviral vectors. **i** HPP MSCs overexpressing *ALPL* or treatment with 0.1 mM metformin, Alizarin Red staining and quantification of mineralized nodules were performed on day 28 after osteogenic induction (OS). Expression levels of Runx2 and OCN were examined by western blotting on day 7 after induction. **j** Oil Red O staining and quantification of fat depots were performed on day 14 after the adipogenic induction (AD). PPAR-γ expression was examined on day 7 after induction by western blotting. Scale bars, 100 μm. (N) Normal control *n* = 5, HPP (hypophosphatasia patient) *n* = 2. The data are presented as the means ± s.d. of each independent experiment performed in triplicate. *P < 0.05, **P < 0.01. **e**-**f**, **i**-**j** One-way analysis of variance (ANOVA). **a**, **d** Unpaired two-tailed Student’s *t*-test.

Given that A*lpl* expression is correlated with the ageing process of murine MSCs and subsequently affects the postnatal bone phenotype, we tested whether *Alpl* regulates the fate of MSCs via ATP metabolism in human MSCs (hMSCs). Two patients with HPP caused by an *ALPL* loss-of-function mutation were confirmed by a clinical examination and DNA sequencing (data not shown). The HPP MSCs had fewer Ki67^+^ cells and LAP2β^+^ cells than the normal controls. However, relatively more SA-β-gal^+^ cells and γ-H2AX^+^ cells and higher expression levels of *p16* and *p53* were detected in the HPP MSCs (Fig. 7a, b), which is consistent with those in the *Alpl*^+/-^ MSCs. Furthermore, the expression levels of CD73 and CD39 did not differ between the normal and HPP hMSCs (Fig. 7c). The elevated levels of extracellular ATP due to the *ALPL* deficiency were also internalized by the hMSCs and inactivated the AMPKα pathway, subsequently causing senescence in the hMSCs (Fig. 7d–h). Finally, similar to the *Alpl*^+/-^ MSCs, the function of the HPP MSCs was more effectively rescued by metformin than the function of the MSCs in the *ALPL* overexpression group (Fig. 7i, j). Collectively, our results indicate that *Alpl* is necessary for the regulation of the lineage differentiation of MSCs and prevention of their senescence.

## Discussion

Our study revealed that *Alpl* orchestrates lineage differentiation and senescence in MSCs through ATP-mediated regulation of AMPKα, thereby playing an important role in bone ageing. Specifically, *Alpl* deficiency results in an excessively high level of extracellular ATP due to enhanced ATP release and reduced ATP hydrolysis, which is subsequently internalized by MSCs, increasing the intracellular ATP level. This elevation inhibits the AMPKα pathway and contributes to the cell fate switch of MSCs. The reactivation of the AMPKα pathway by metformin rescues endogenous MSC function and prevents premature bone ageing in *Alpl*^+/-^ mice (Supplementary Fig. 9).

Heterozygous *Alpl*^+/-^ mice survive normally and exhibit a 50% decrease in TNSALP activity and expression.^46^ Previous studies have revealed that *Alpl*^+/-^ mice do not exhibit significant radiographic evidence of skeletal disease.^46^ However, we observed that the 4-month-old *Alpl*^+/-^ mice exhibited not only severe bone mass loss but also a marked marrow fat gain. Furthermore, the expression of the ageing-related genes *p16* and *p53* was also increased in the *Alpl*^+/-^ mice, indicating that the *Alpl* deficiency led to the premature ageing of bone. A recent study revealed that a targeted deletion of *Alpl* in both mouse OBs (Col1a1-Cre) and mesenchymal (Prx1-Cre, used to delete genes from mesenchymal progenitor cells) resulted in skeletal defects, including hypomineralization, hyperosteoidosis, rachitic changes, bone deformities and deterioration.^47^ Importantly, we observed that the senescent cells in the BM were mostly colocalized with MSCs rather than with mature OBs. Thus, we inferred that the *Alpl* deficiency-induced bone ageing phenotype may occur via the targeting of endogenous MSCs rather than mature OBs.

In bone, *Alpl* is localized on the entire cell surface of preosteoblasts^48^ and has long been used as an osteoblastic marker.^16,49^ MSCs are common progenitors of OBs and adipocytes in BM and abundantly express *Alpl*,^13^ which is consistent with our results. However, the mechanism by which *Alpl* regulates the function of MSCs remains elusive. *Alpl* is expressed in one-cell-stage embryos^50^ and has been proposed to ensure that ES cells are maintained in an undifferentiated state,^51^ suggesting that *Alpl* plays one or several functions in these undifferentiated cells and/or during their multipotential differentiation.^52^ We observed that *Alpl*^+/-^ MSCs exhibited enhanced adipogenic differentiation at the expense of osteogenic differentiation. In addition, the expression levels of the ageing-related genes *p16* and *p53* were increased after the induction of *Alpl* deficiency. More convincingly, the normal MSCs were changed into a diseased-cell phenotype after the downregulation of *Alpl*, whereas the function of diseased MSCs was rescued by *Alpl* overexpression. To further validate the role of *Alpl* in the fate switch of murine MSCs, we tested the characteristics of cultured MSCs from two patients with HPP. Although HPP is caused by an *ALPL* loss-of-function mutation, the patients are usually characterized by defective bone mineralization and a deficiency in serum and bone ALP activity.^5–8^ Here, the two patients displayed significant lower ALP activity and TNSALP expression both in MSCs and serum (data not shown). Moreover, the *ALPL* level in the hMSCs regulated their senescence and lineage differentiation, which is consistent with the observations in the *Alpl*^+/-^ MSCs. Given that *Alpl* is localized in the cell membrane of MSCs, we discovered that *Alpl* is necessary for the cell fate choice of MSCs, subsequently affecting the phenotype of postnatal bone.

hMSCs have been reported to spontaneously release ATP,^19^ and ATP levels may dramatically increase when the microenvironment is changed.^25,53^ Moreover, extracellular ATP can, in turn, affect the function of MSCs, including inhibiting proliferation,^19^ stimulating migration^54^ and regulating differentiation.^55,56^ Here, we found that *Alpl* deficiency leads to an elevated extracellular ATP concentration primarily due to increased ATP release and decreased ATP hydrolysis. This excessive extracellular ATP subsequently performs sequential important functions in MSCs, including elevating the intracellular ATP level, inactivating the AMPKα pathway and impairing cell function. However, whether and how MSCs take up extracellular ATP remain unclear. Previous studies have reported that extracellular ATP regulates cell function by activating ionotropic P2X and metabotropic P2Y receptors.^39–41^ Additionally, a recent study showed that extracellular ATP in the form of nonhydrolyzable fluorescent NHF-ATP is internalized by macropinocytosis.^26^ However, in this study, we observed that neither macropinocytosis nor purinergic receptor signaling could account for the intracellular ATP elevation. In contrast, the extracellular ATP directly entered the cytoplasm of MSCs as evidenced by the regular ATP and radioactive ATP-γ-P^32^ analyses. Collectively, we revealed that *Alpl* could simultaneously orchestrate ATP release and internalization and has an important impact on cell function.

Metformin, which is a first-line drug used for the treatment of type 2 diabetes, has been shown to successfully extend longevity and lifespan in experimental mice.^57^ Therefore, the use of metformin as an anti-ageing drug has been recently suggested based on its wide application in clinical practice, well-known pharmacokinetics and acceptable toxicity.^45,57^ In our study, the metformin treatment exhibited a stronger capacity to rescue the function of *Alpl*^+/-^ MSCs both in vitro and in vivo than the recovery of the *Alpl* level. We believe that although the elevation in intracellular ATP was rescued by the overexpression of *Alpl*, the impaired intracellular pathways in the MSCs were still incompletely recovered, partially accounting for the weaker therapeutic effect. Some previous studies have reported that metformin may protect against fragility fracture.^21^ However, other studies have revealed that although a minor effect of metformin on bone metabolism cannot be excluded, the actions of this drug on the bone were not sufficient to modify the incidence of fractures.^21^ Here, we found that injections of metformin into the BM cavity could successfully rescue the impaired function of endogenous MSCs and prevent premature bone ageing in *Alpl*^+/-^ mice. We believe that the different therapeutic effect compared with that reported in previous studies may be due to the murine model and different routes of metformin administration.

Collectively, we revealed a previously unrecognized role of *Alpl* in the prevention of bone ageing via ATP-mediated MSC functional alterations and that pathway-guided metformin treatment may provide an effective therapy for *Alpl*-deficient bone ageing. Further studies are needed to explore the mechanism by which *Alpl* regulates the ability of MSCs to release ATP. Furthermore, because many organs in the *Alpl*^+/-^ mice manifested ageing characteristics (data not shown), systematic injections of metformin in *Alpl*^+/-^ mice and confirmation of the changes in other organs may be needed in future experiments.

## Materials and methods

### Mice

Female *Alpl*^+/+^, *Alpl*^+/-^ (B6.129S7-Akp2^tm1Sor^/J, pure C57BL/6J genetic background) and *Alpl*^Cre/+^; Rosa^26mTmG/+^ (Stock No. 007676) mice were purchased from Jackson Laboratories (Bar Harbor, ME, USA). Female immunocompromised nude (CAnN.Cg-Foxn1nu/CrlVr) 2- and 24-month-old mice were purchased from Vital River Laboratory Animal Technology Co. Ltd. (Beijing, China). Female SAMPR1 and SAMP6 mice were purchased from Peking University Laboratory Animal Center. All procedures involving animals were approved by the Animal Use and Care Committee of the Fourth Military Medical University (license number: SYXK 2012–0023).

Primers used for genotyping include the following: oIMR0137 – 5ʹ-CCGTGCATCTGCCAGTTTGAGGGGA-3ʹ – Mutant; oIMR0138 – 5ʹ-CTGGCACAAAAGAGTTGGTAAGGCAG-3ʹ – Wild-type; oIMR0139 – 5ʹ-GATCGGAACGTCAATTAACGTCAAT-3ʹ – Common.

### ALP activity assay

The serum and intracellular ALP activities were estimated by using a kit from NJJCBIO Company (NJJCBIO, Nanjing, China) according to the manufacturer’s instruction. The final ALP activity was expressed as micromoles of p-nitrophenol release.

The ALP activity in serum can be calculated as follows: ALP activity (U⋅L^–1^) = A/V/T, where A refers to the amount of pNP generated by the samples (in μmol); V refers to the volume of sample added to the assay well (in L); and T refers to the reaction time (in minutes).

The ALP activity in cells is calculated as follows: ALP activity (U⋅g^–1^) = A/V/T, where A refers to amount of pNP generated by the samples (in μmol); V refers to the number of samples added to the assay well (in g); and T refers to the reaction time (in minutes).

### Immunofluorescent staining

The bones were fixed in 4% paraformaldehyde for 24 h at 4 °C, decalcified in 10% EDTA (pH 7.4), embedded in or tissue-freezing medium (Leica), and sectioned into 10 μm sections. For the immunofluorescence assay, heat-induced antigen retrieval was performed with sodium citrate buffer (10 mmol⋅L^–1^ sodium citrate, 0.05% Tween 20, pH 6.0) before the bone sections were blocked with 10% normal serum containing 1% bovine serum albumin (BSA) in Tris-buffered saline and Tween 20 (TBST) (pH 7.6) for 2 h at room temperature; then, the sections were incubated overnight at 4 °C with primary antibodies against Sca-1/Ly6A/E (Abcam, Cambridge, MA, USA,1:100), p16^INK4α^ (Abcam, 1:100), OCN (Santa Cruz, Texas, CA, USA, 1:50), PPAR-γ (Abcam, Cambridge, MA, USA, 1:50) and phospho-AMPKα (Cell Signaling, Boston, MA, USA, 1:50) for 2 h and subsequently incubated with secondary antibodies. The positive cells were examined under a laser scanning confocal microscope (Olympus FluoViem FV 1000, Tokyo, Japan). The quantitative histomorphometric analysis was conducted with Image-Pro Plus software.

### Immunohistochemistry analysis

At the time of sacrifice, the femora were resected and fixed in 4% paraformaldehyde, decalcified with 10% EDTA (pH 7.0) and embedded in paraffin. Then, 6-μm thick longitudinal sections were prepared for TNSALP immunohistochemistry. We incubated the sections with a primary antibody against mouse TNSALP (R&D Systems, Minneapolis, MN, USA, 1:100) overnight at 4 ℃ and subsequently with secondary antibodies. The detection of the immunoreaction was achieved using a streptavidin-horse radish peroxidase system (Dako, Carpinteria, CA, USA). The quantitative histomorphometric analysis was conducted with Image-Pro Plus software.

### Senescence-associated β-galactosidase staining

The femora were resected and fixed in 4% paraformaldehyde, decalcified with 10% EDTA (pH 7.0) and embedded in paraffin. Then, 6-μm thick longitudinal sections and MSCs seeded in a 24-well plate for 24 h were prepared for the SA-β-gal staining. We used a staining kit (Cell Signaling). Briefly, we collected the bone slices and MSCs and then rinsed the sample once with 1 × phosphate-buffered saline (PBS; 2 mL or a 35 mm well plate or matched volume of media). Then, 1 mL of 1 × Fixative Solution was added to each 35 mm well, and the samples were allowed to fix for 10–15 min at room temperature. The samples were rinsed twice with 1 × PBS. Then, 1 mL β-gal staining solution was added to each 35 mm well, and the sample was incubated at 37 ℃ at least overnight in a dry incubator. The SA-β-gal^+^ cells were stained blue under a microscope. The samples were analyzed by Image-Pro Plus software.

### μCT analysis

The mice femora were scanned with an Inveon μCT system (Siemens AG, Germany). The cross-sectional volumetric bone mineral density (BMD) was measured at the right femur mid-diaphysis. Using two-dimensional images, a region of interest in the secondary spongiosa was manually drawn near the endocortical surface, and cancellous bone morphometric parameters, including the BMD and BV/TV, were assessed.

### Oil red O staining

To assess the fat tissue in the trabecular areas, the mice femora were fixed in 4% paraformaldehyde and decalcified with 10% EDTA (pH7.0), followed by cryosectioning. Then, 10-μm thick sections were stained with Oil Red O for 10–20 min at room temperature, and the positive areas were quantified under a microscope and shown as a percentage of the total area. Then, the sections were washed with 60% isopropanol and twice with PBS. All reagents used for the Oil Red O staining were purchased from Sigma-Aldrich (St. Louis, MO, USA).

### Bone histomorphometric analysis

To examine the new bone formation rate, the mice received a double injection of calcein intraperitoneally (10 mg⋅kg^−1^ body weight; Sigma) 14 d and 2 d prior to euthanasia. The tibiae and femora were isolated, fixed in 95% ethanol, and embedded in methyl methacrylate. A microtome was used to prepare 50-μm longitudinal sections. The bone dynamic histomorphometric analyses of BFR/BS were performed using Image-Pro Plus software under a fluorescence microscope (Leica, Wetzlar, Germany, #DMI6000B).

### TRAP staining

To perform the TRAP staining, decalcified femora sections were fixed with a mixture of 3% formaldehyde, 67% acetone and 25% citrate solution and stained for TRAP using a commercially available kit (Sigma). Five random fields of view per section were captured under a microscope to quantify the number of TRAP^+^ cells.

### ELISA assay

We collected WT and *Alpl*^+/-^ mouse serum and performed a CTX-1 ELISA analysis by using mouse EIA kits (BOSTER, Wuhan, China). We performed all ELISA assays according to the manufacturers’ instructions.

### Levamisole injection in vivo

Levamisole (10 mg⋅kg^−1^ body weight dissolved in 15 μL NaCl, Sigma) was injected into the femora bone marrow cavity of 4-month-old *Alpl*^+/-^ mice three times per week for 1 month. The control mice received a comparable volume of NaCl (*n* = 8 per group). All mice were harvested at day 30 after the injections for analysis.

### Isolation of mouse BM MSCs

The mouse BM cells (2 × 10^7^–3 × 10^7^) were flushed from the long bones with 3% fetal bovine serum (FBS) in PBS. A single-cell suspension of all nuclear cells was obtained by passing all BM cells through a 70-μm cell strainer (Bioscience, Dümmer, Germany). Then, 2.5 × 10^5^ cells per square centimeter were seeded into 10-cm culture dishes (Corning, Lowell, MA, USA) and incubated at 37 ℃ in 5% CO_2_. After 48 h, the cultures were washed with PBS to eliminate the non-adherent cells. The attached cells were cultured for 10–15 d with α-modified essential medium (α-MEM; Gibco-BRL, Gaithersburg, MA, USA) supplemented with 20% FBS (Gibco-BRL), 2 mmol⋅L^−1^ L-glutamine, 100 U⋅mL^−1^ penicillin, and 100 mg⋅mL^−1^ streptomycin (Invitrogen, Gaithersburg, MD, USA). The cell culture protocol and surface marker identification have been described in our previous studies^16,58^ (Supplementary Table 1).

### qRT-PCR analysis

The total RNA was isolated from the cells, BM and other soft tissues by using RNAiso plus (TaKaRa, Tokyo, Japan) according to the manufacturer’s instructions. The mRNA was converted to complementary DNA, and quantitative reverse transcriptase-PCR (qRT-PCR) detection was carried out by PrimeScript^TM^ RT master mix (TaKaRa, RR036A) and SYBR Premix Ex Taq^TM^II (TaKaRa). A CFX96 Trademark Real-time PCR detection system (Bio-Rad, Richmond, CA, USA) was used for the detection. The expression levels of *p16*^*INK4A*^, *p53* and *TERT* (TaKaRa) were examined.

### Flow cytometric analysis

The BM cells were flushed, collected in α-MEM with 2% FBS, diluted to 1 × 10^6^ cells in 400 μL of medium and incubated with anti-mouse CD45-APC (eBioscience, San Diego, CA, USA), CD31-PE (eBioscience), Ly-6A/E (Sca-1) FITC (eBioscience), and CD73-PE (eBioscience) antibodies for 30 min at 4 ℃. Then, the cells were sorted using fluorescence-activated cell sorting (FACS) Aria model II (BD Bioscience), and the analysis was performed with FlowJo software version 7.6.

For the identification of the cell surface markers of MSCs, 5 × 10^5^ WT and *Alpl*^+/-^ MSCs were incubated with Ly-6A/E (Sca-1) FITC (eBioscience), CD73-PE (eBioscience), CD90.1/Thy1.1-FITC (eBioscience), CD34-PE (Biolegend), CD45-APC (eBioscience) and TNSALP (R&D systems) antibodies for 30 min on ice. The samples were analyzed using FACS Aria model II, and the analysis was performed with FlowJo software, version 7.6.

Both 1 × 10^6^ WT and *Alpl*^+/-^ MSCs cultured for 72 h were collected in 1.5 mL Eppendorf (EP) tubes and centrifuged at 300 × *g* for 5 min at room temperature. The cells were washed three times with PBS, followed by resuspension in 400 µL binding buffer (eBioscience), and divided equally into two new tubes. One sample was used as a negative control, whereas 10 µL Annexin V-FLUOS and 10 µL propidium iodide were added to the other sample, which was incubated at room temperature for 15 min. The samples were analyzed using a FACS Aria model II, and the analysis was performed with FlowJo software version 7.6.

### Osteogenic and adipogenic differentiation assays

The WT and *Alpl*^+/-^ MSCs were incubated with osteogenic medium (100 nmol⋅L^−1^ dexamethasone, 50 mg⋅mL^−1^ ascorbic acid and 1 mmol⋅L^−1^ b-glycerophosphate) (Sigma) for 21 d according to the manufacturer´s instructions. To assess osteogenic differentiation, the cells were fixed with 60% isopropanol and stained with 1% Alizarin Red (Sigma). The expression levels of Runx2 and OCN were assayed by western blotting on day 7 after the osteogenic induction.

The WT and *Alpl*^+/-^ MSCs were cultured with adipogenic medium (0.5 mmol⋅L^–1^ methylisobutylxanthine, 0.5 mmol⋅L^−1^ hydrocortisone and 60 mmol⋅L^–1^ indomethacin; Sigma) for 14 d. The intracellular lipid accumulation was detected by staining with Oil Red O solution. PPARγ expression was assayed by western blotting on day 7 after the adipogenic induction.

### In vivo bone formation assay

After culturing for 3 d, approximately 5 × 10^6^ MSCs were mixed with 40 mg hydroxyapatite/tricalcium phosphate (HA/TCP) ceramic particles (Sigma) and implanted into subcutaneous pockets on the backs of 8-week-old immunocompromised mice. The control group was implanted on the other side of the same host. The implants were removed 2 months after transplantation, fixed with 4% paraformaldehyde and decalcified with buffered 10% EDTA (pH 7.0). For the histological analyses, the sections were stained with hematoxylin and eosin (HE) or Masson’s trichrome (BaSO Diagnostic Inc., Guangdong, China). The sections were analyzed by Image-Pro Plus software.

### Western blotting analysis

The MSCs were harvested in RIPA lysis buffer (Beyotime Co., Shanghai, China). After the whole-cell protein extracts were quantified by a bicinchoninic acid (BCA) assay, the extracts were separated on NuPAGE 10%–12% polyacrylamide gels, transferred onto polyvinylidene fluoride (PVDF) membranes (Millipore, Billerica, MA, USA), blocked in 5% BSA in TBST, and hybridized with antibodies against β-actin (Abcam, 1:4 000), TNSALP (Abcam; R&D Systems, 1:500), p16^INK4α^ (Abcam, 1:1 000), p53 (Cell Signaling, 1:1 000), Runx2 (Cell Signaling, 1:1 000), OCN (Santa Cruz, 1:800), PPAR-γ (Abcam, 1:300), AMPKα (Cell Signaling, 1:1 000), phospho-AMPKα (Thr172) (Cell Signaling, 1:1 000), ACC (Cell Signaling, 1:1 000), phospho-ACC (Cell Signaling, 1:1 000), CD73 (Cell Signaling, 1:1 000) and CD39 (Proteintech, 1:800). β-Actin was used as a loading control. The signals were revealed after incubation with a secondary antibody (CABIO Biotech., Beijing, China) coupled to peroxidase by using electrochemiluminescence (ECL).

### Lentiviral vector construction and transduction

To construct the lentiviral vector, mouse *Alpl* and human *ALPL* were amplified by PCR from human genomic DNA. The PCR product was digested with *Age*I and *Eco*RI restriction enzymes, inserted into the pLko.1 vector (Addgene), digested with *Bam*HI and *Xho*I and inserted into the pLenti 6.3/v5-DEST vector (Addgene). The inserted fragments were verified by Sanger sequencing. A lentiviral construct containing a scrambled *Alpl/ALPL* sequence was used as a negative control. The lentivirus was produced by co-transfecting 293T cells with the transfer vector and two packaging vectors (i.e., psPAX2 and pMD2G). The virus was subsequently purified by ultracentrifugation. The MSCs were plated in six-well plates and transduced with the lentiviral constructs and 10 µg⋅mL^−1^ polybrene (Sigma). The primers used to construct the lentiviral vectors of *Alpl* and *ALPL* are listed in Supplementary Table 2.

### Transfection assay

siRNA duplex oligonucleotides against mouse AMPKα1/2 were obtained from Santa Cruz. Non-targeting control siRNAs (Santa Cruz) were used as negative controls. In addition, the siRNAs were transfected into the cells at a final concentration of 50 nM using siPORT *NeoFX* (Invitrogen). The medium was replaced after 8 h.

### PPi measurements

The extracellular and intracellular PPi concentrations were measured by a spectrophotometric method using an EnzChek^®^ Pyrophosphate Detection Kit (Molecular Probes, Madison, USA). Briefly, we collected the culture medium or cell lysates and then added 10 μL samples or standard substance into a final 100 μL reaction mixture in a 96-well plate. The reaction mixture was incubated for another 30–60 min at 22 ℃, and the absorbance was read at 360 nm. Use a Pi-free laboratory is extremely important for processing the collected samples.

### ALP inhibitor and mimic treatment

To examine the effect of TNSALP on the ATP level, we treated the WT MSCs with 100 μmol⋅L^−1^ ALP inhibitor (Levamisole, Sigma) and the *Alpl*^+/-^ MSCs with 1 U⋅mL^−1^ TNSALP from porcine kidney (Sigma). The cell lysates were prepared, and all ATP levels were examined by an Enhanced ATP Assay Kit (Beyotime).

### Measurement of extracellular and intracellular ATP concentration

The WT and *Alpl*^+/-^ cell medium and cell samples with or without lentiviral vector transfection were collected by ATP lysis buffer and centrifuged at 12 000 × *g* at 4 ℃ for 5 min; then, the supernatants and medium of the cells were examined by an Enhanced ATP Assay Kit (Beyotime). The intracellular ATP concentration results were corrected with the total protein levels of each sample.

The WT and *Alpl*^+/-^ MSCs were treated with 10 μmol⋅L^−1^ ATP in the presence or absence of 10 μmol⋅L^−1^ Oligomycin A (SelleckChem, Houston, Texas, USA), 50 μmol⋅L^−1^ EIPA (APEXBIO, Houston, Texas, USA), 30 μmol⋅L^−1^ PPADS (Sigma) or 100 μmol⋅L^−1^ Suramin (Sigma) for 1 h; then, the cells were collected by ATP lysis buffer and centrifuged at 12 000 × *g* at 4 ℃ for 5 min. The supernatants of the cells were examined by an Enhanced ATP Assay Kit (Beyotime). The intracellular ATP concentration results were corrected with the total protein levels of each sample.

To examine the effect of ATP or ATP-apyrase on the intracellular ATP level, we treated the WT MSCs with 10 μmol⋅L^−1^ ATP (Sigma) and the *Alpl*^+/-^ MSCs with 2 U⋅mL^−1^ ATP-apyrase (Sigma). The cell lysates were prepared, and all ATP concentration examinations were performed according to the manufacturer’s instructions. The results were corrected with the total protein levels of each sample.

### Radioactive ATP assay

According to the experimental design, the cells were treated with 10 µmol⋅L^−1^ ATP, which contained 2 μCi ATP-γ-^32^P (3 000 Ci per mmol, BLU002A250UC, PerkinElmer, Waltham, MA, USA), and reacted at 37 °C in 5% CO_2_ for 1 h. Then, the ATP solution was removed, and the cells were thoroughly washed with PBS. In addition, the cells were lysed. The samples were mixed with OptiPhase Supermix Cocktail (1200–439, PerkinElmer) and incubated for 10 min, and the radioactive signals were examined by a Luminescence Counter (MicroBeta JET, PerkinElmer) for 60 s. This study was performed to ascertain that ATP directly enters the cytoplasm of MSCs.

### Metformin treatment in vitro

In total, 3 × 10^5^ MSCs were plated into each well of a six-well plate and cultured. After the cells reached 70%–80% confluency, the cells were cultured in osteogenic/adipogenic differentiation medium containing 0.1 mmol⋅L^–1^ Metformin (Sigma) as described above, and the medium was changed every 2 d. On day 7, we harvested the cells and subjected them to western blotting assays of OB- and adipocyte-related genes. On day 21 and 14, we performed Alizarin Red and Oil Red O staining.

### Metformin injection in vivo

Metformin (60 mg⋅kg^−1^ body weight dissolved in 15 μL NaCl, Sigma) was injected into the femora BM cavity of 4- and 12-month-old *Alpl*^+/-^ mice every 2 weeks for 1 month (total of two injections). The control mice received a comparable volume of NaCl (*n* = 8 per group). All mice were harvested on day 30 after the injection for analysis.

### HPP subjects

Two HPP patients (male) aged 8 and 13 years were treated by the Affiliated Hospital of Fourth Military Medical University for osteodynia and missing teeth. The healthy human BM samples were collected from five teenagers aged 10–13 years (male) who underwent alveolar bone cleft repair by auto-ilium transplantation.

### Isolation of human BM MSCs

The cells were purified from the BM by the Percoll density gradient centrifugation method and cultured in α-MEM supplemented with 10% FBS (Gibco-BRL), 2 mmol⋅L^−1^ L-glutamine (Invitrogen), 100 U⋅mL^−1^ penicillin and 100 mg⋅mL^−1^ streptomycin (Invitrogen) at 37 °C in 5% CO_2_. The third passage MSCs were subjected to induction of osteogenic and adipogenic differentiation.

### Statistics

The data are presented as the mean ± s.d. The data were assessed for normal distribution and similar variance among the groups before further analysis. We used unpaired, two-tailed Student’s *t-*tests for comparisons between two groups and one-way analysis of variance (ANOVA) with Bonferroni for multiple comparisons. All experiments were repeated more than three times, and representative experiments are shown. *P*-values <0.05 were considered significant. All experimental group sizes were chosen to ensure adequate statistical power despite the highly variable nature of the studies performed. No animals or samples were excluded from analysis, and the animals were randomly assigned to groups in the studies.

## Electronic supplementary material

Revised supplementary figures

IRB review

Revised full uncutted gels

Supplementary data
