# Correction: *Alpl* prevents bone ageing sensitivity by specifically regulating senescence and differentiation in mesenchymal stem cells

**DOI:** 10.1038/s41413-020-00107-z

**Published:** 2020-07-28

**Authors:** Wenjia Liu, Liqiang Zhang, Kun Xuan, Chenghu Hu, Shiyu Liu, Li Liao, Bei Li, Fang Jin, Songtao Shi, Yan Jin

**Affiliations:** 1 MS-State Key Laboratory & National Clinical Research Center for Oral Diseases & Shaanxi International Joint Research Center for Oral Diseases, Center for Tissue Engineering, School of Stomatology, Fourth Military Medical University, Xi’an, China; 2 Xi’an Institute of Tissue Engineering and Regenerative Medicine, Xi’an, China and 3Department of Anatomy and Cell Biology, School of Dental Medicine, University of Pennsylvania, Philadelphia, PA USA

**Keywords:** Pathogenesis, Bone

Correction to: *Bone Research*https://www.nature.com/articles/s41413-018-0029-4, published online 11 September 2018

During a re-read of our article [1], previously published in *Bone Research*, we noticed that Fig. 3b (second panel of line 4), which presented LAP2β immunostaining of first-passage MSCs from *Alpl*^+/−^ mice at 4 months, was consistent to Fig. 7a (fourth panel of line 2), which was presented the LAP2β immunostaining of HPP MSCs. All the authors agree to rectify this mistake by rearranging Fig. 7 below.

**Fig. 7:**
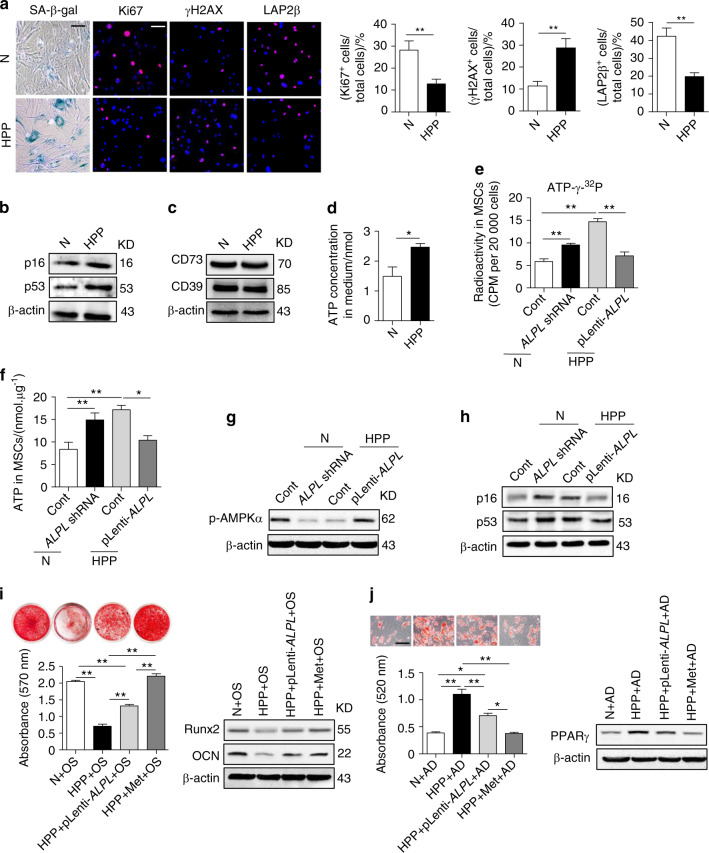
*Alpl* also controls the differentiation and senescence of human MSCs via ATP-mediated inactivation of the AMPKα pathway. **a** SA-β-gal staining and Ki67, γH2AX and LAP2β immunostaining of third-passage MSCs from normal controls and HPP patients. Quantification of Ki67^+^, γH2AX^+^ and LAP2β^+^ is indicated in the right panel. Scale bars: 50 μm. **b** Expression levels of ageing-specific genes in normal and HPP yiMSCs were examined by western blotting. Scale bars, 50 μm. **c** Expression levels of CD73 and CD39 in normal and HPP MSCs were examined by western blotting. **d** Extracellular ATP concentrations in normal and HPP MSC medium were examined by a regular ATP concentration assay. **e** Intracellular radioactivity was examined after a 1-h treatment with ATP-γ-^32^P in different lentiviral vector transduction groups. **f** Intracellular ATP concentrations were assayed 48 h after the transduction of different lentiviral vectors. **g** Western blotting analysis of p-AMPKα expression in normal control and the ALPL shRNA, HPP control and pLenti-ALPL groups. **h** Expression levels of p16 and p53 were assayed 48 h after the transduction of different lentiviral vectors. **i** HPP MSCs overexpressing ALPL or treatment with 0.1 mmol·L^−1^ metformin, Alizarin Red staining and quantification of mineralized nodules were performed on day 28 after osteogenic induction (OS). Expression levels of Runx2 and OCN were examined by western blotting on day 7 after induction. **j** Oil Red O staining and quantification of fat depots were performed on day 14 after the adipogenic induction (AD). PPAR-γ expression was examined on day 7 after induction by western blotting. Scale bars, 100 μm. (N) Normal control *n* = 5, HPP (hypophosphatasia patient) *n* = 2. The data are presented as the means ± s.d. of each independent experiment performed in triplicate. *P < 0.05, **P < 0.01. **e**–**f**, **i**–**j** One-way analysis of variance (ANOVA). **a**, **d** Unpaired two-tailed Student’s t-test.

This correction does not affect the results or conclusions of the above paper. We apologize for this typographical error and any inconvenience caused.
