# Supplementary material for: Alpl prevents bone ageing sensitivity by specifically regulating senescence and differentiation in mesenchymal stem cells
Source: Bone Res. 2018 Sep 11;6:27. doi: 10.1038/s41413-018-0029-4 (PMC6131243; doi:10.1038/s41413-018-0029-4)
Supplement: Supplementary file 1 — Revised supplementary figures [file 41413_2018_29_MOESM1_ESM.pdf]

Alpl prevents bone aging sensitivity through specific regulation of senescence and differentiation of MSC

Wenjia Liu[1,2], Liqiang Zhang[1,2], Kun Xuan[1], Chenghu Hu[2], Shiyu Liu[1], Li Liao[2], Bei Li[1], Fang Jin[1], Songtao Shi[3], Yan Jin[1,2,*]

1. MS-State Key Laboratory & National Clinical Research Center for Oral Diseases & Shaanxi International Joint Research Center for Oral Diseases, Center for Tissue Engineering, School of Stomatology, Fourth Military Medical University, Xi'an, 710032, China.
2. Xi' an Institute of Tissue Engineering and Regenerative Medicine，Xi' an, 710032, China.
3. Department of Anatomy and Cell Biology, School of Dental Medicine, University of Pennsylvania, Philadelphia, PA, USA.
These authors contributed equally to this work.
*Correspondence: Yan Jin, PhD. Phone: +86-29-84776471, Fax: +86-29-83218039.
E-mail: yanjin@fmmu.edu.cn or yanjinfmmu@139.com.

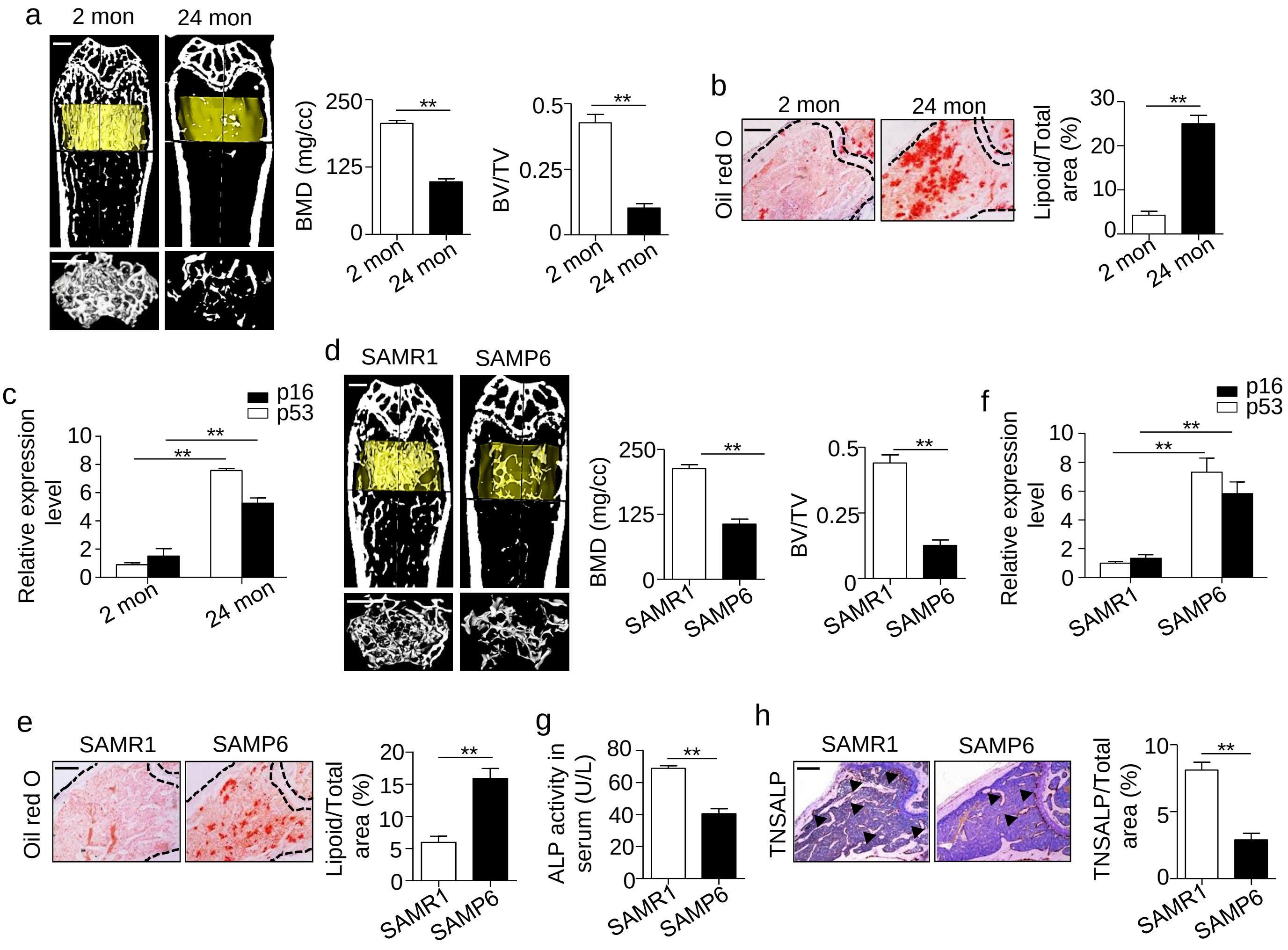

Supplementary Fig. 1 *Alpl* expression also declines in the BM of senescence-accelerated mice model, related to Fig 1. (**a** and **d**) μCT images and the quantification of BMD and BV/TV. Scale bars, 1 mm. (**b** and **e**) Oil red O staining images and quantitative analysis of the area of adipose tissue over the total area of the femoral diaphysis. Scale bars, 500 μm. (**c** and **f**) The expression levels of aging-specific genes *p16* and *p53* were examined via qRT-PCR. (**g**) The serum ALP activities in SAMR1 and SAMP6 mice were examined by the ALP activity assay. (**h**) Immunohistochemical analysis of TNSALP (brown) in the femoral diaphysis. Quantification of TNSALP^{+} area is indicated in the right pannel. Scale bars, 500 μm. n = 6 for all groups. SAMR1, control for SAMP6. SAMP6, senescence-accelerated mouse prone 6. The data are presented as means ± s.d. of each experiments performed in triplicate. *$P < 0.05$, **$P < 0.01$. Unpaired two-tailed Student's t-test.

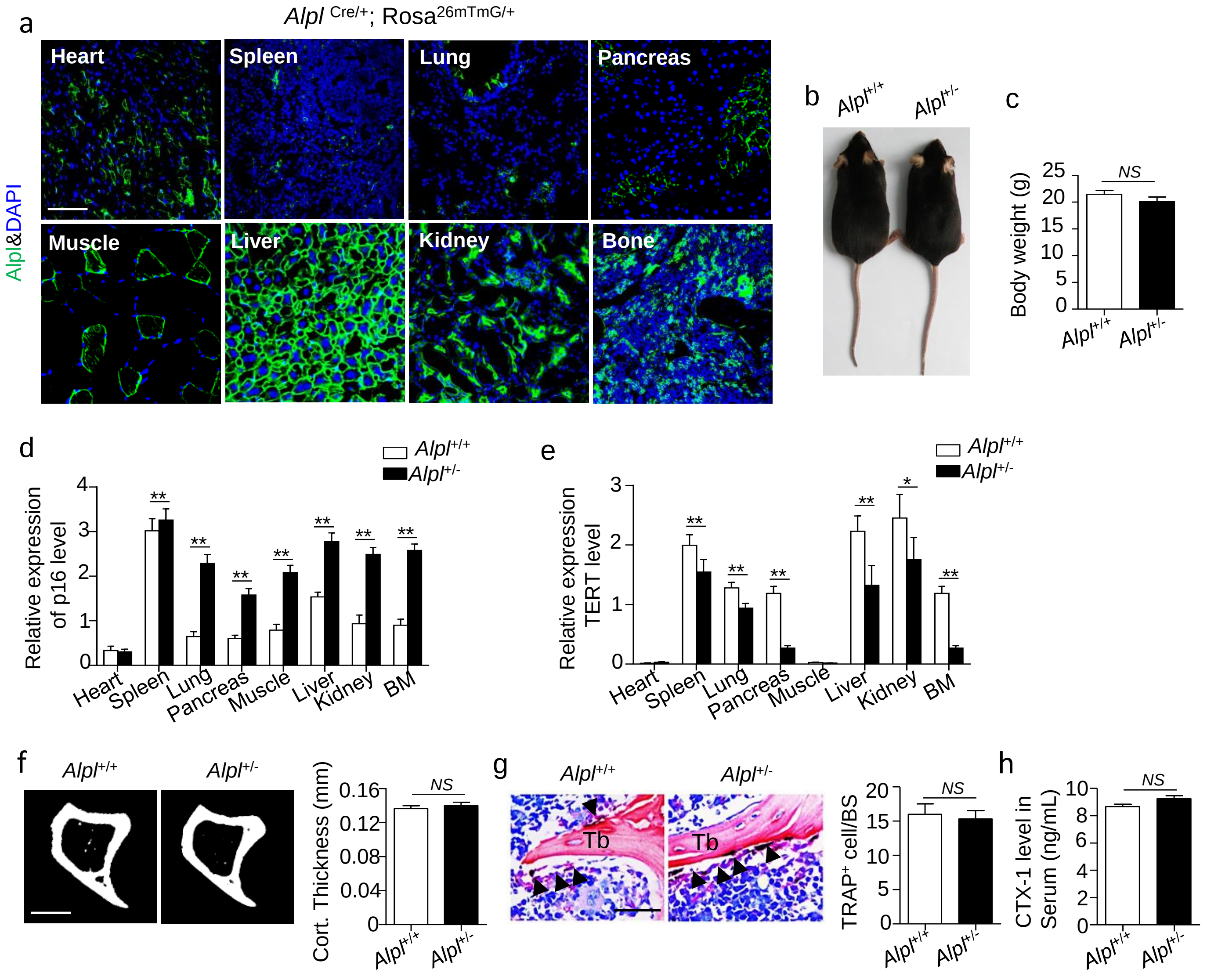

Supplementary Fig. 2 Heterozygous *Alpl*$^{+/-}$ mice exhibit premature bone aging characteristics independent of osteoclastogenesis, related to Fig 2. (**a**) Representative image of immunostaining analysis showed that *Alpl* (green) in various tissues and organs of 2-month-old *Alpl* $^{Cre/+}$; Rosa$^{26mTmG/+}$ mice. Scale bar, 50 μm. (**b** and **c**) The appearance and body weight of *Alpl*$^{+/+}$ and *Alpl*$^{+/-}$ mouse was almost the same. (**d** and **e**) The expression levels of *p16* and *TERT* in 4-month-old *Alpl*$^{+/+}$ and *Alpl*$^{+/-}$ mice were examined via qRT-PCR. (**f**) μCT images of cortical bone and quantification of cortical thickness (Ct.Th). Scale bars, 1 mm. (**g**) TRAP staining and quantitative analysis of osteoclasts number in femoral diaphysis. Scale bars, 50 μm. (**h**) ELISA analysis of CTX-1 in *Alpl*$^{+/+}$ and *Alpl*$^{+/-}$ mice serum. n = 6 for all groups. The data are presented as means ± s.d. of each experiments performed in triplicate. *$P < 0.05$, **$P < 0.01$, *NS,* not significant. Unpaired two-tailed Student's t-test.

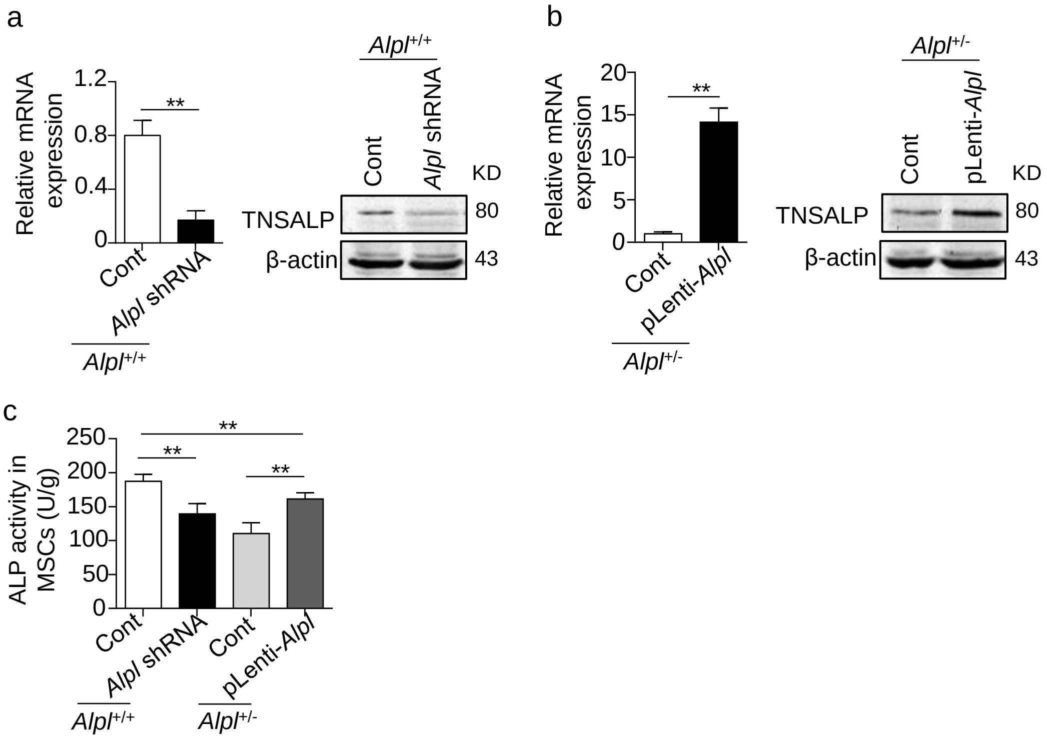

Supplementary Fig. 3 The efficiency of lentivirus transduction, related to Fig 3, 4. (**a** and **b**) The efficiency of lentiviral vector on knockdown or overexpressing *Alpl* was confirmed by qRT-PCR and western blotting analysis. (**c**) The ALP activities were examined after 48 h of transduction by ALP activity assay. n = 6 for all groups. The data are presented as means ± s.d. of each independent experiments performed in triplicate. **P* < 0.05, ***P* < 0.01. a and b, Unpaired two-tailed Student's t-test. c, One-way analysis of variance (ANOVA).

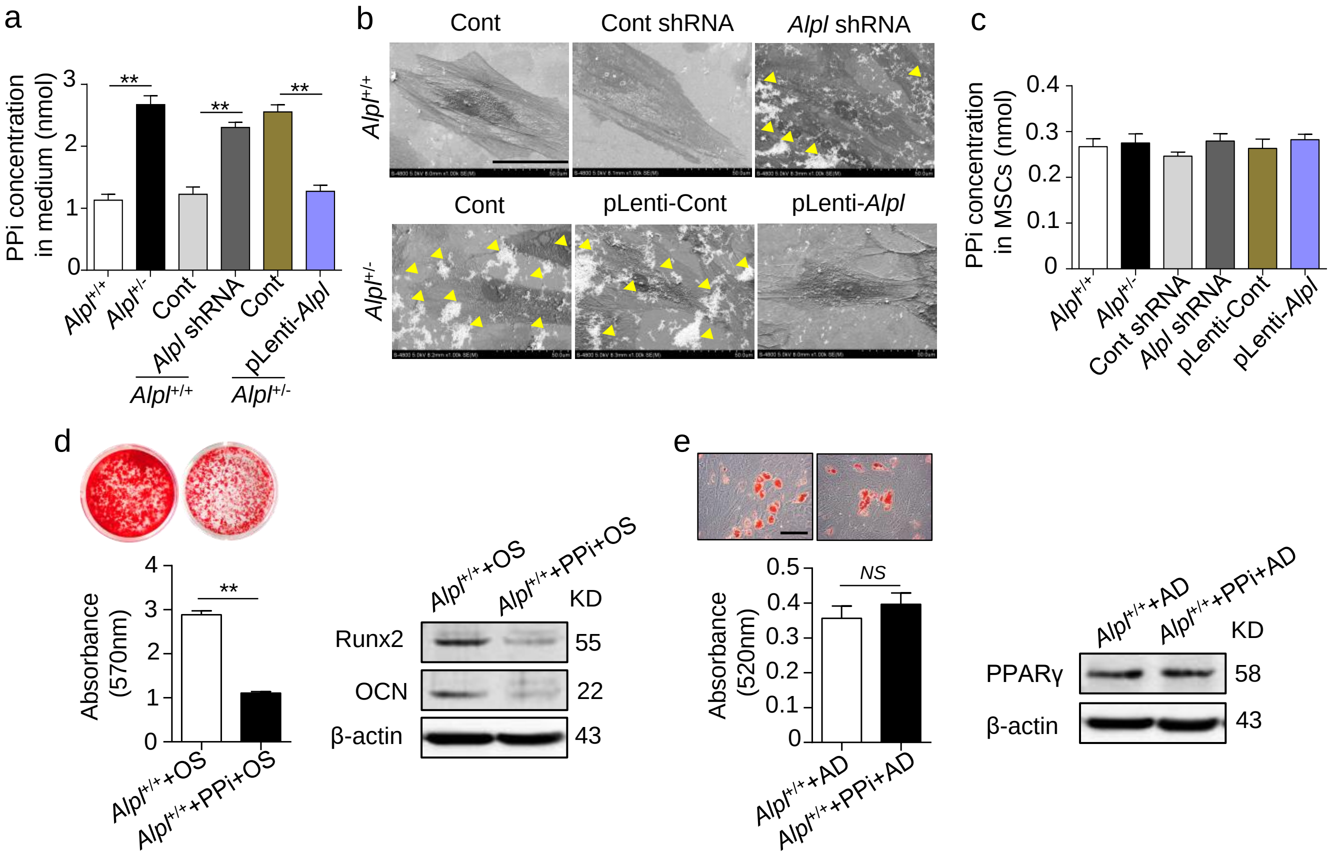

Supplementary Fig. 4 *Alpl* deficiency induces excessive PPi accumulated in extracellular of MSCs, which only inhibits osteogenesis, related to Fig 4. (**a** and **c**) The extracellular and intracellular PPi concentrations were assayed after 48 h of transducing different lentiviral vectors. (**b**) Scanning electron microscope images for examining extracellular PPi accumulation. Scale bars, 50 μm. (**d**) *Alpl*$^{+/+}$ MSCs were treated with 10 μM PPi, alizarin red staining and quantification of mineralized nodules were performed at day 21 after osteogenic induction (OS). Runx2 and OCN expression levels were examined at day 7 after induction by western blotting. (**e**) Oil red O staining and quantification of fat depots were performed at day 14 after adipogenic induction (AD). PPAR-γ expression was examined at day 7 after induction by western blotting. Scale bars, 200 μm. n = 6 for all groups. The data are presented as means ± s.d. of each independent experiments performed in triplicate. *P < 0.05, **P < 0.01, a and c, One-way analysis of variance (ANOVA). d and e, Unpaired two-tailed Student's t-test.

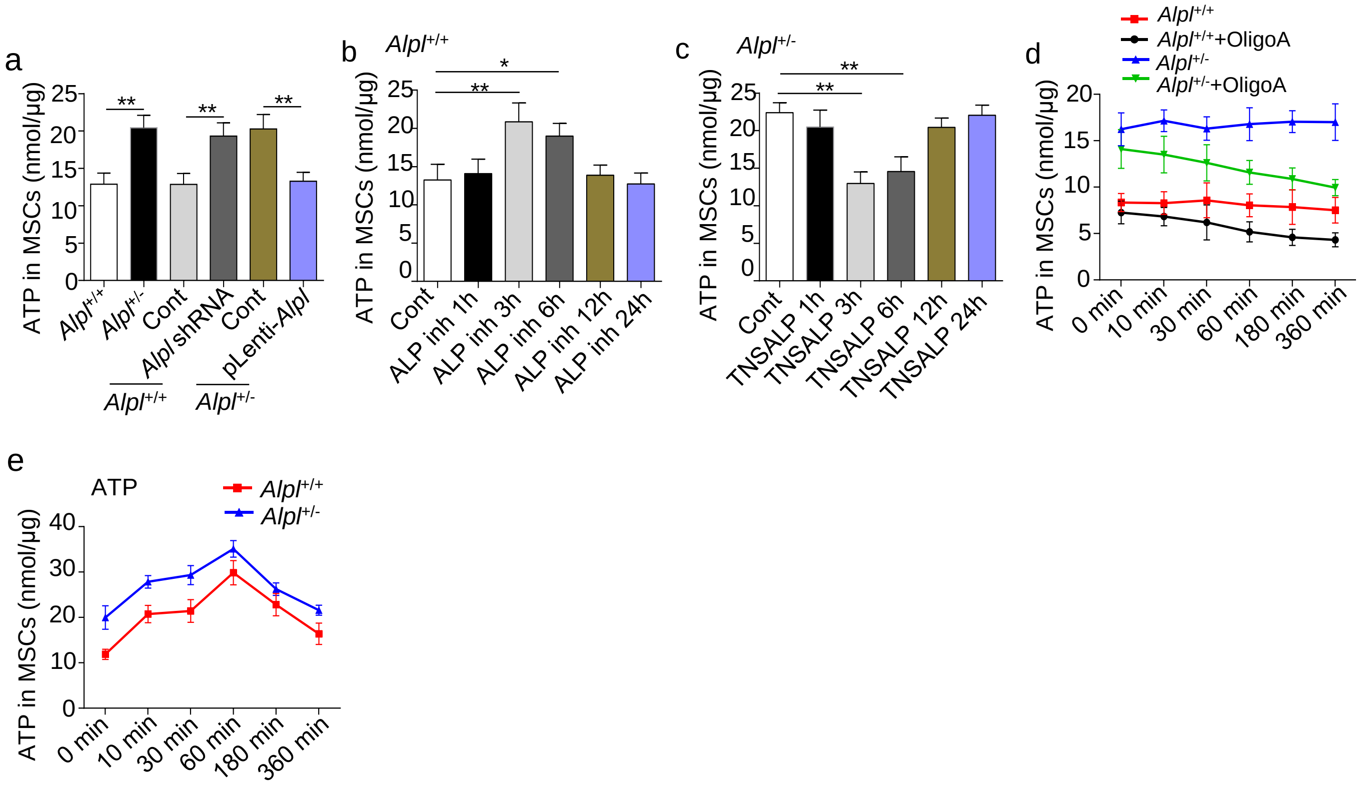

Supplementary Fig. 5 The intracellular ATP concentration is varied with TNSALP level, related to Fig 4. (**a**) The intracellular ATP concentrations were assayed after 48 h of transducing different lentiviral vectors. (**b** and **c**) The intracellular ATP concentrations in *Alpl*$^{+/+}$ MSCs treated with 100 μM ALP inhibitor (inh, levamisole) and *Alp*l$^{+/-}$ MSCs treated with 1 U/ml TNSALP were examined at 0 h, 1 h, 3 h, 6 h, 12 h and 24 h. (**d**) *Alpl*$^{+/+}$ and *Alpl*$^{+/-}$ MSCs treated with 10 μM oligomycin A, the intracellular ATP concentrations were assayed at 0 min, 10min, 30min, 60min, 120min, 180min and 360 min after treatment. (**e**) *Alpl*$^{+/+}$ and *Alpl*$^{+/-}$ MSCs treated with 10 μM ATP, the intracellular ATP concentrations were assayed at 0 min, 10min, 30min, 60min,120min,180min and 360 min after treatment. n = 6 per group. The data are presented as means ± s.d. of each independent experiments performed in triplicate. *P < 0.05, **P < 0.01. a-d, One-way analysis of variance (ANOVA). e, Unpaired two-tailed Student's t-test

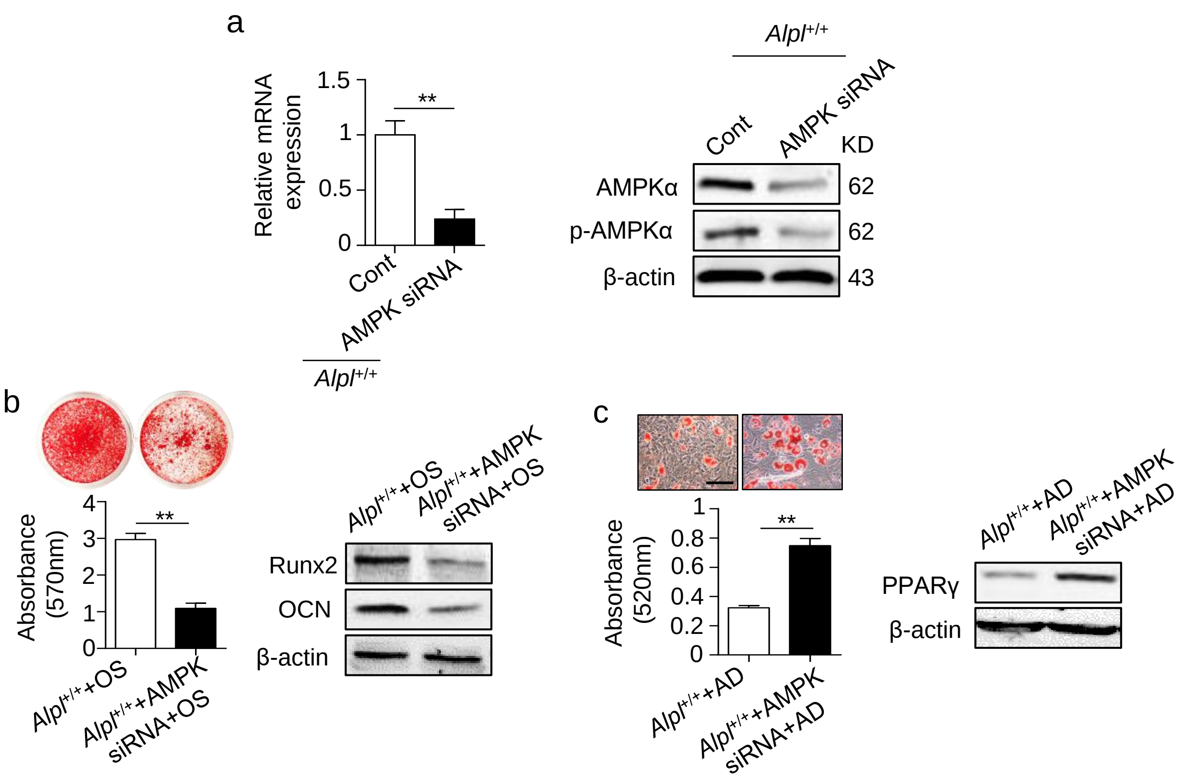

Supplementary Fig. 6 The AMPKα pathway promotes osteogenic differentiation at the expense of adipogenic differentiation of MSCs, related to Fig 5. (**a**) The efficiency of AMPKα siRNA was confirmed by qRT-PCR and western blotting analysis. (**b**) After transfection with AMPKα siRNA, alizarin red staining and quantification of mineralized nodules were performed at day 21 after osteogenic induction (OS). Runx2 and OCN expression levels were examined at day 7 after induction by western blotting. (**c**) Oil red O staining and quantification of fat depots were performed at day 14 after adipogenic induction (AD). PPAR-γ expression was examined at day 7 after induction by western blotting. Scale bars, 100 μm. n = 6. The data are presented as means ± s.d. of each independent experiments performed in triplicate. **P* < 0.05, ***P* < 0.01. Unpaired two-tailed Student's t-test.

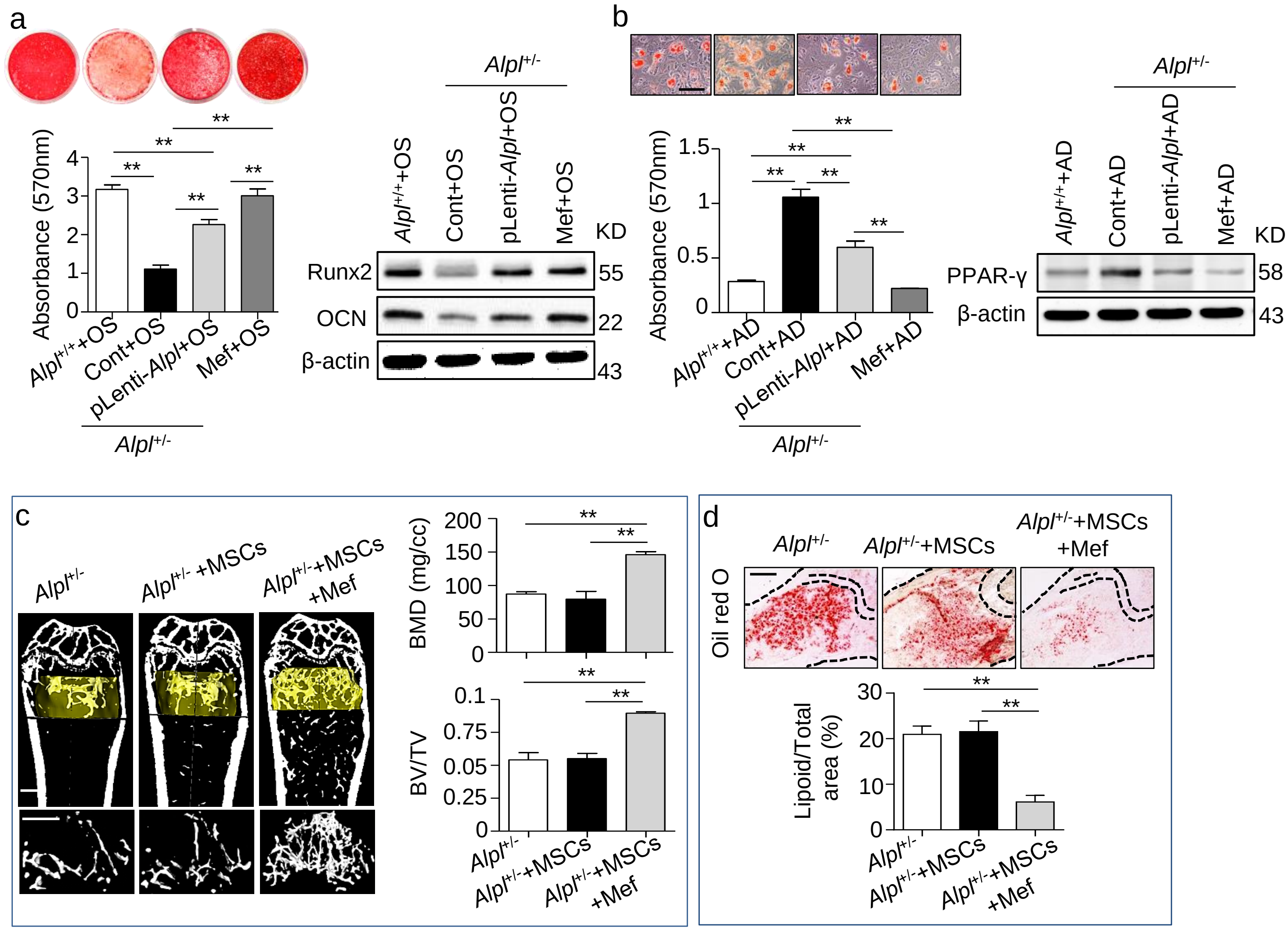

Supplementary Fig. 7 Pathway-guided reactivation of the AMPKα by metformin is effective to rescue the function of *Alpl*$^{+/-}$ MSCs both *in vitro* and *in vivo,* related to Fig 6. (**a**) *Alpl*$^{+/-}$ MSCs treated with *Alpl* lentivirus and 0.1 mM metformin, alizarin red staining and quantification of mineralized nodules were performed at day 21 after osteogenic induction (OS). Expression levels of Runx2 and OCN were examined through western blotting at day 7 after induction. (**b**) Oil red O staining and quantification of fat depots were performed at day 14 after adipogenic induction (AD). PPAR-γ expression was examined at day 7 after induction by western blotting. Scale bars, 100 μm. (**c**) *Alpl*$^{+/-}$ MSCs were treated with 0.1 mM metformin for 3 d and subsequently injected into *Alpl*$^{+/-}$ mice, 1 × 10^5 MSCs per 10 g body weight. μCT images and quantification of BMD and BV/TV. Scale bars, 1 mm. (**d**) Oil red O staining images and quantitative analysis of the area of adipose tissue over the total area of the femoral diaphysis. Scale bars, 500 μm. n = 6 for a and b; n = 8 for c and d. The data are presented as means ± s.d. of each independent experiments performed in triplicate. **P* < 0.05, ***P* < 0.01. One-way analysis of variance (ANOVA).

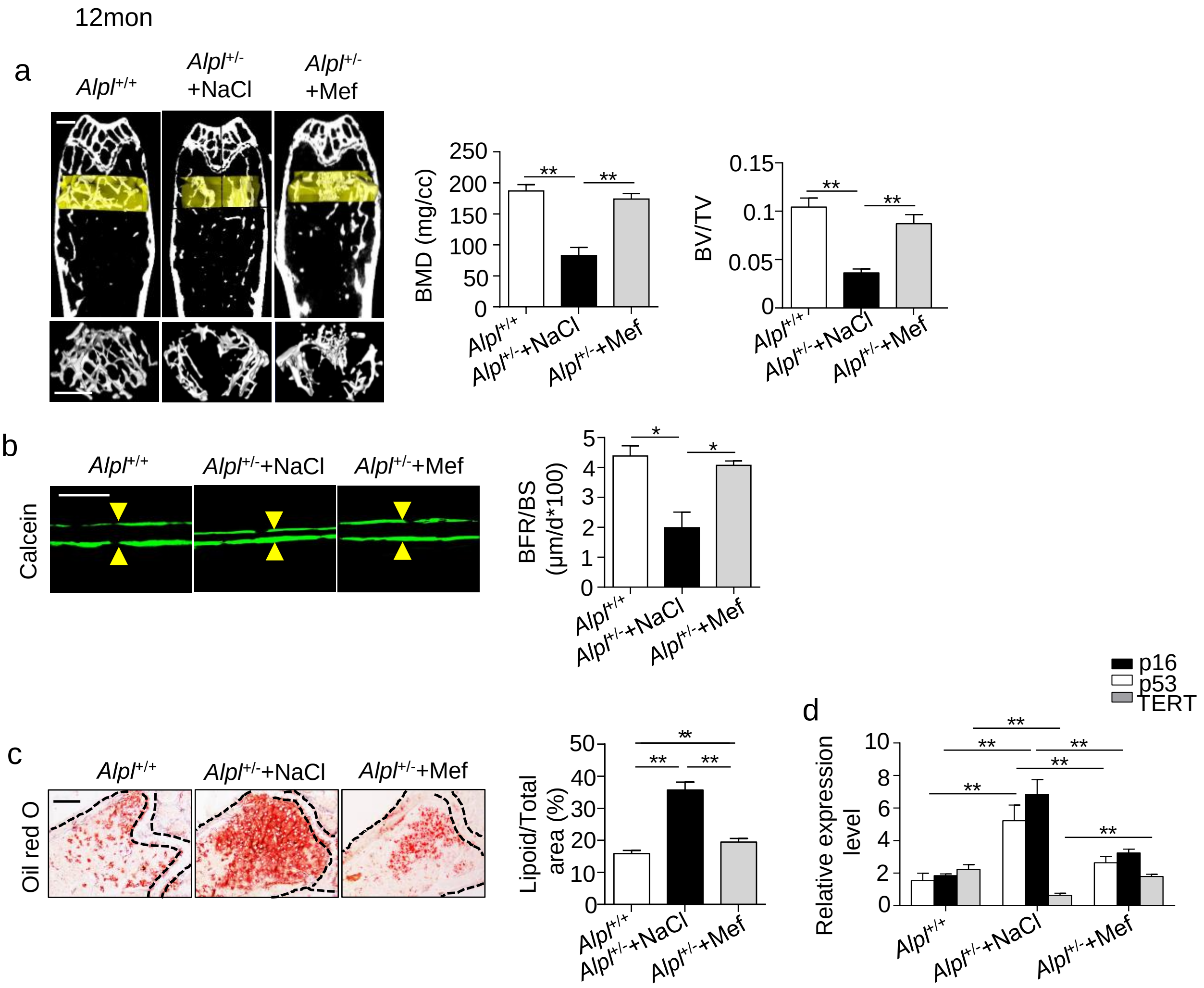

Supplementary Fig. 8 Metformin treatment also shows therapeutic effect on preventing bone aging in 12-month-old *Alpl*$^{+/-}$ mice. We injected metformin into the femoral bone marrow cavity of *Alpl* $^{+/-}$ mice every two weeks at 60 mg/kg for 1 month (totally 2 injections), and NaCl was used as a control. (**a**) μCT images and quantification of BMD and BV/TV. Scale bars, 1 mm. (**b**) Images of calcein double labeling of trabecular bone with quantification of BFR/BS. Scale bars, 50 μm. (**c**) Oil red O staining images and quantitative analysis of the area of adipose tissue over the total area of the proximal femoral diaphysis. Scale bars, 500 μm. (**d**) The expression levels of aging-specific genes were examined via qRT-PCR. n = 6 for all groups. The data are presented as means ± s.d. of each independent experiments performed in triplicate. *P < 0.05, **P < 0.01. One-way analysis of variance (ANOVA).

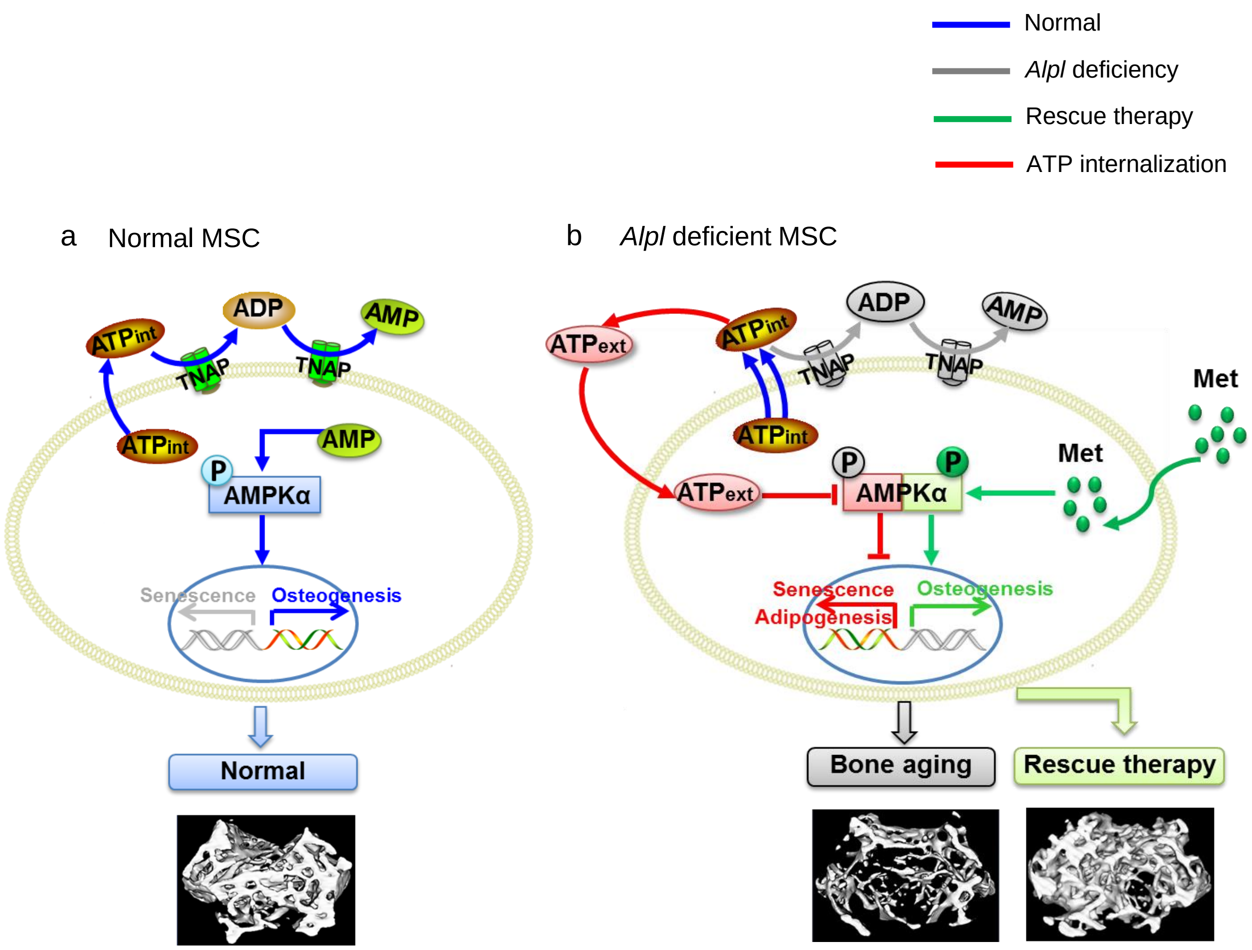

Supplementary Fig 9. Schematic diagram depicting how *Alpl* regulates the MSC fate change and pathway-guided therapeutic method. (**a**) TNSALP sequentially hydrolyzed ATP into AMP and activated the AMPKα pathway that resulted in osteogenesis and prevented senescence of MSCs. (**b**) When *Alpl* deficiency, more ATP was released from MSCs and less ATP was hydrolyzed, which caused excessive ATP accumulated extracellularly. Importantly, these ATP were, in turn, internalized by MSCs and subsequently inducing an elevation of intracellular ATP, inactivation of AMPKα pathway and the fate change of MSCs. Metformin treatment rescued the MSC function and prevented the premature bone aging in *Alpl*$^{+/-}$ mice via reactivation of the AMPKα pathway.
