# Supplementary figures and images for: Alpl prevents bone ageing sensitivity by specifically regulating senescence and differentiation in mesenchymal stem cells
Source: Bone Res. 2018 Sep 11;6:27. doi: 10.1038/s41413-018-0029-4 (PMC6131243; doi:10.1038/s41413-018-0029-4)

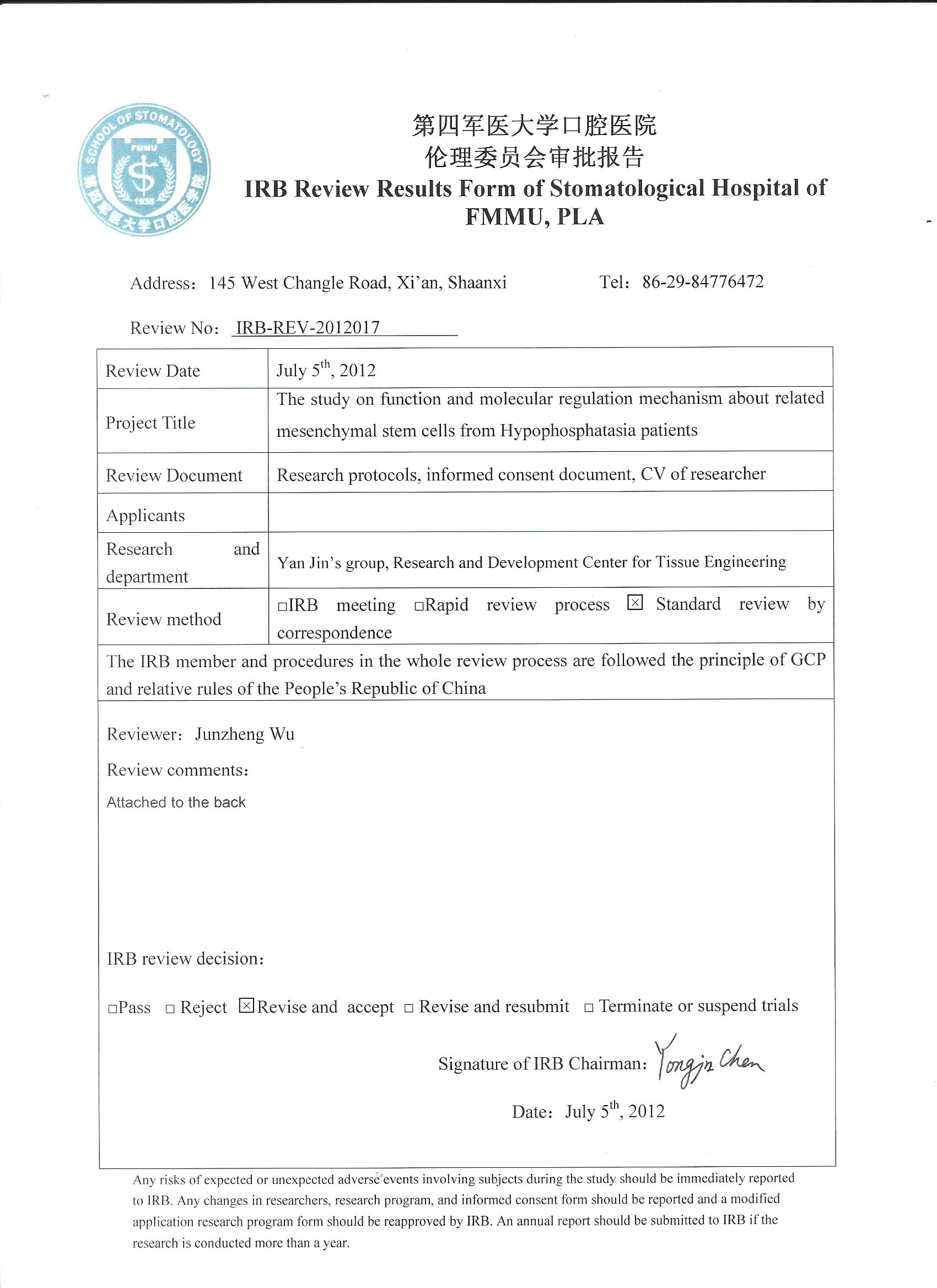

Supplement: Supplementary file 2 — IRB review [file 41413_2018_29_MOESM2_ESM.jpg]

**Fig. 1**

**c**

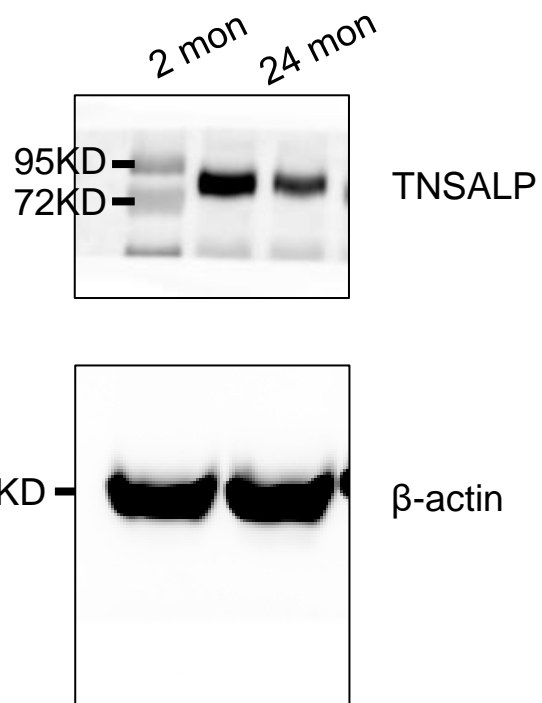

**g**

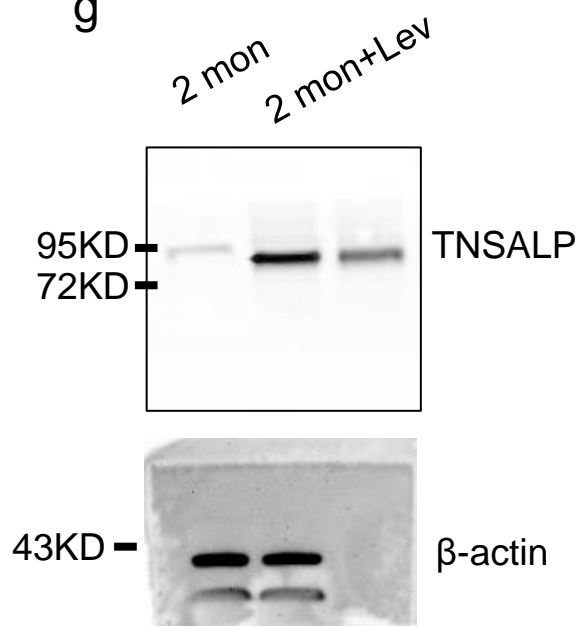

**Fig. 2**

j

$Alpl^{+/+}$      $Alpl^{+/-}$

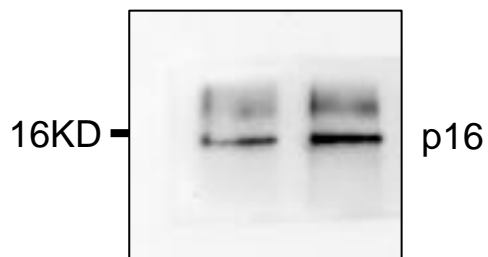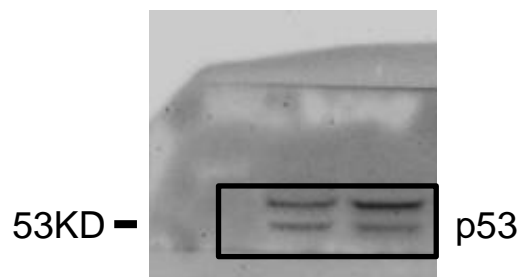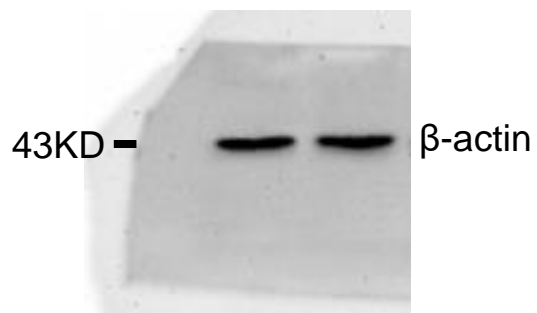

**Fig. 3**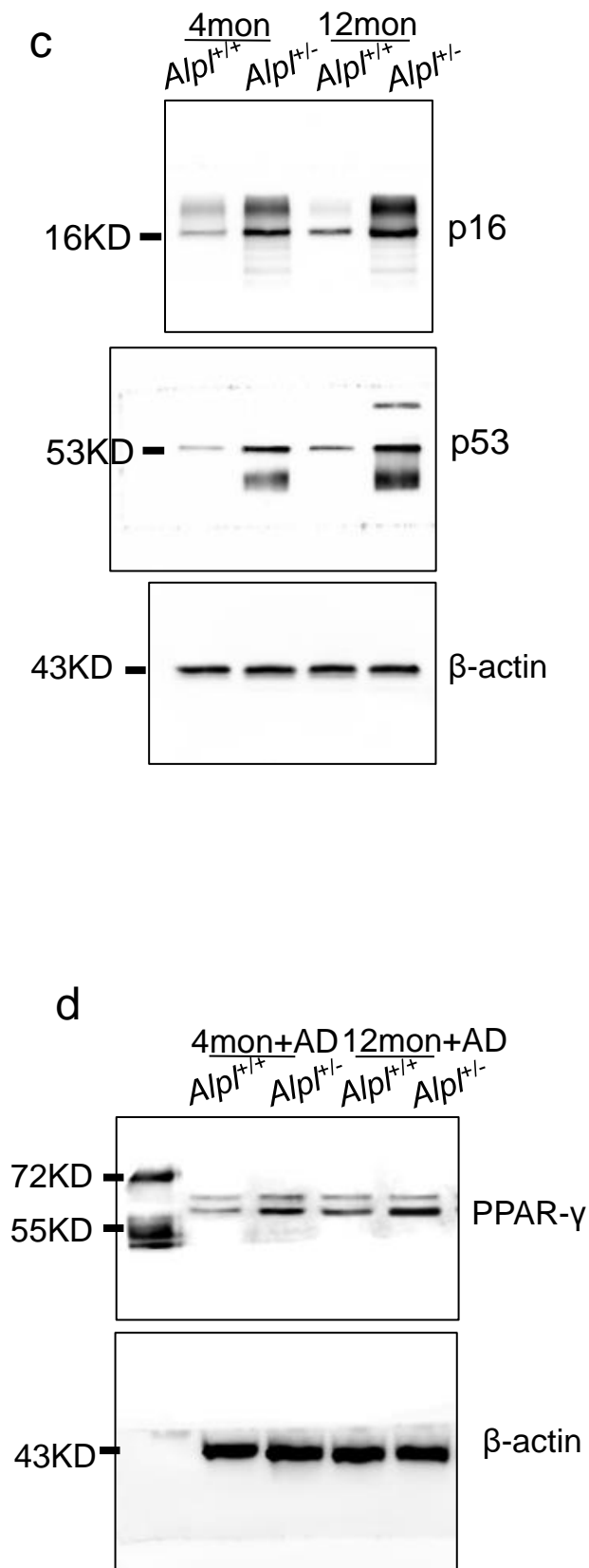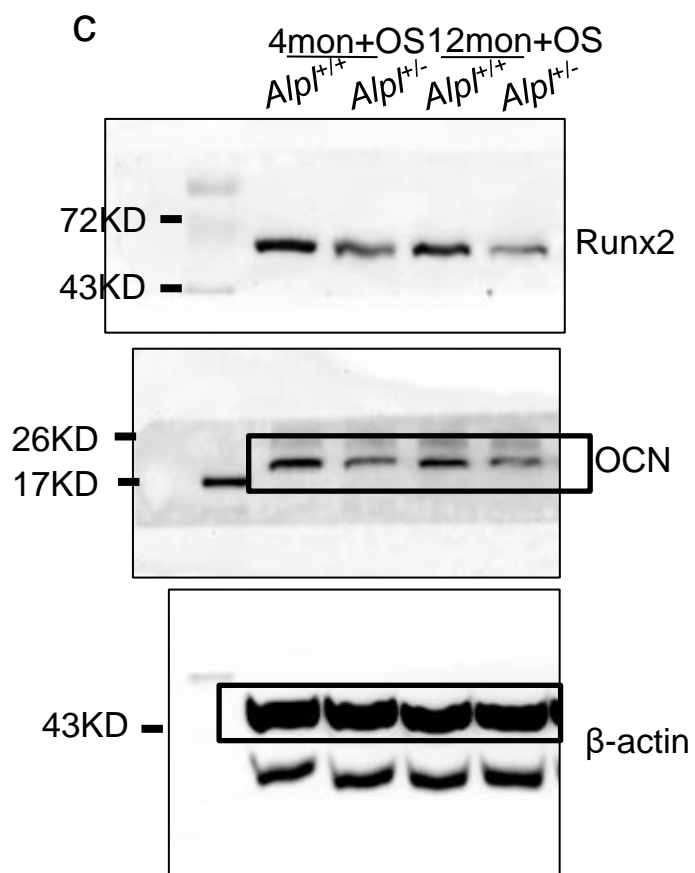

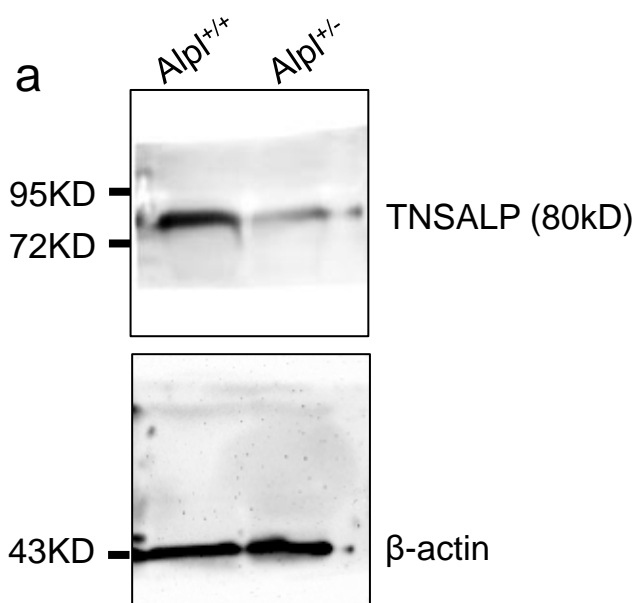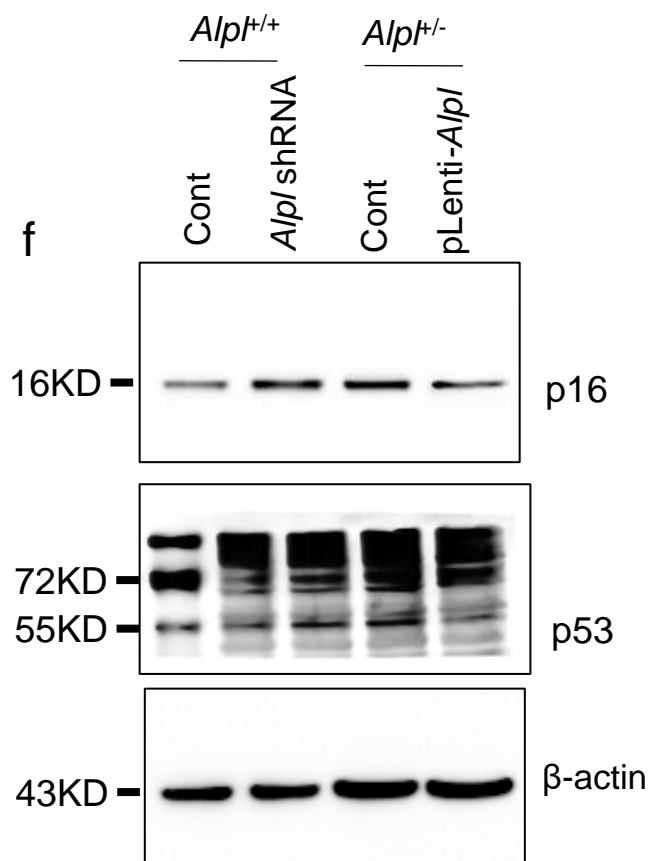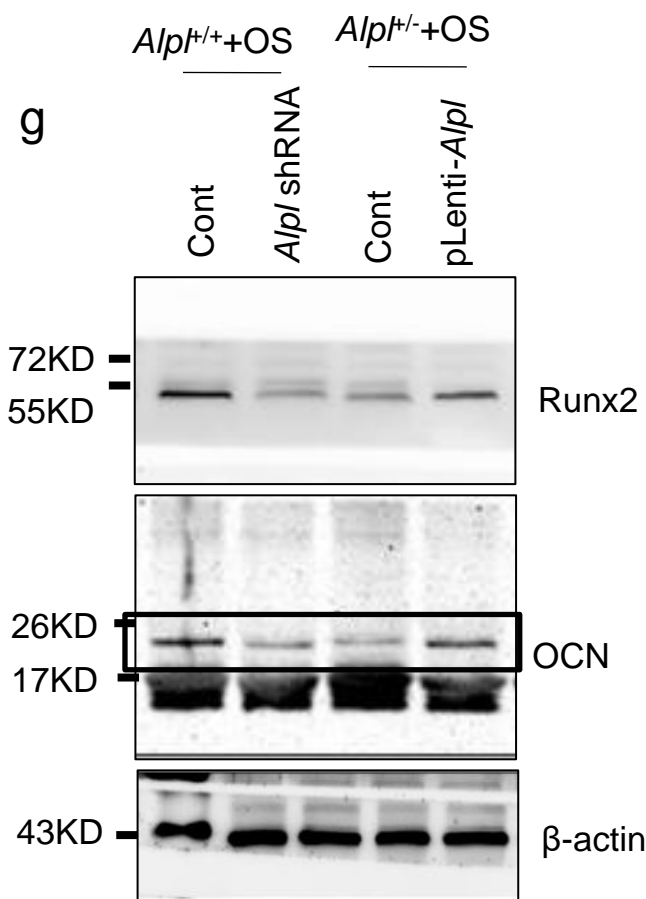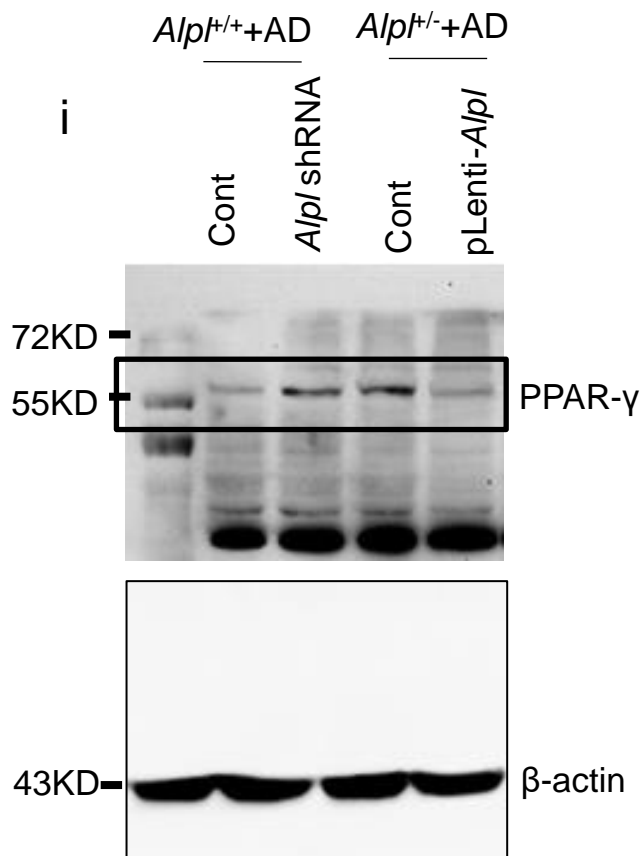

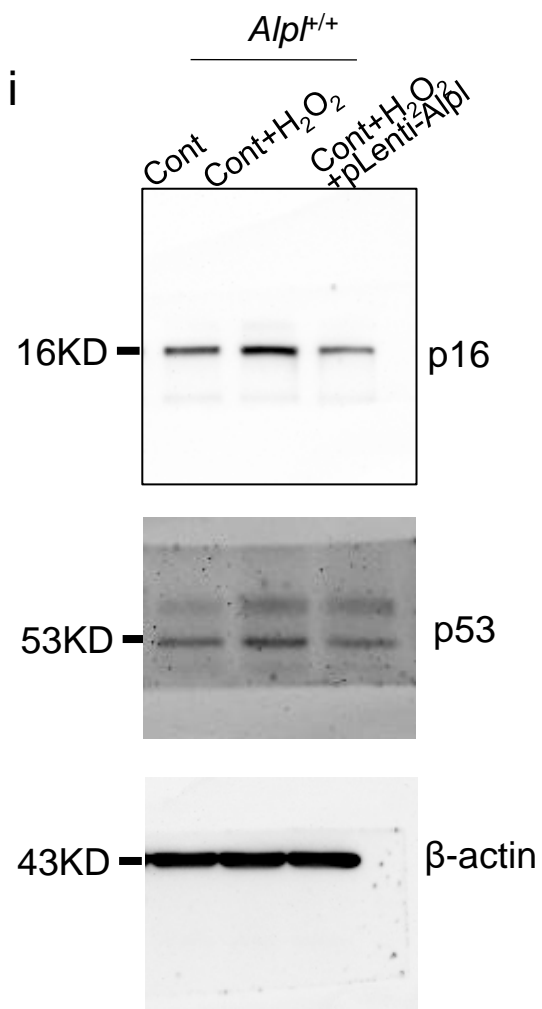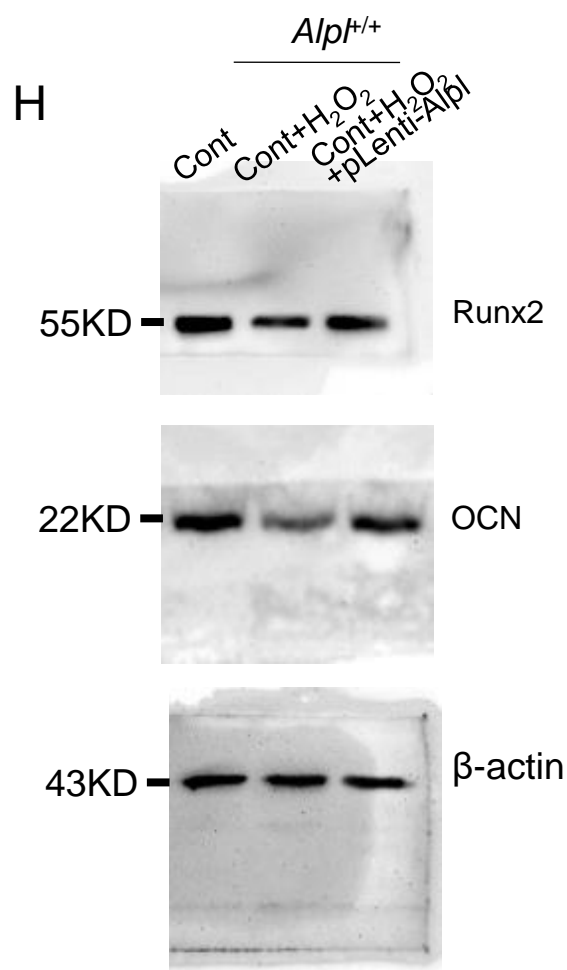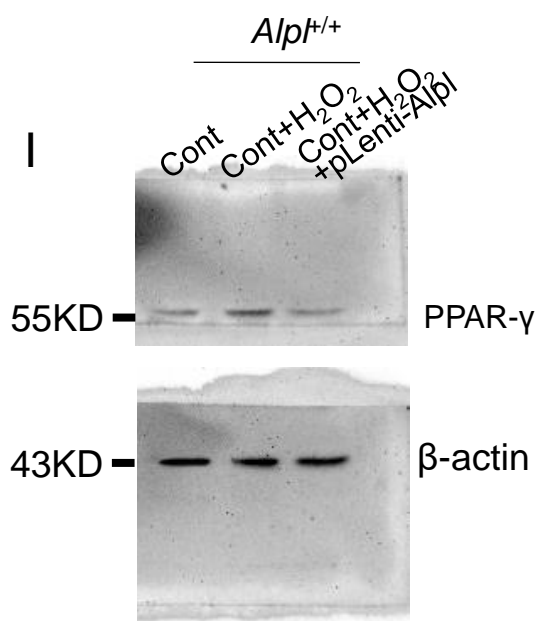

**Fig. 4**

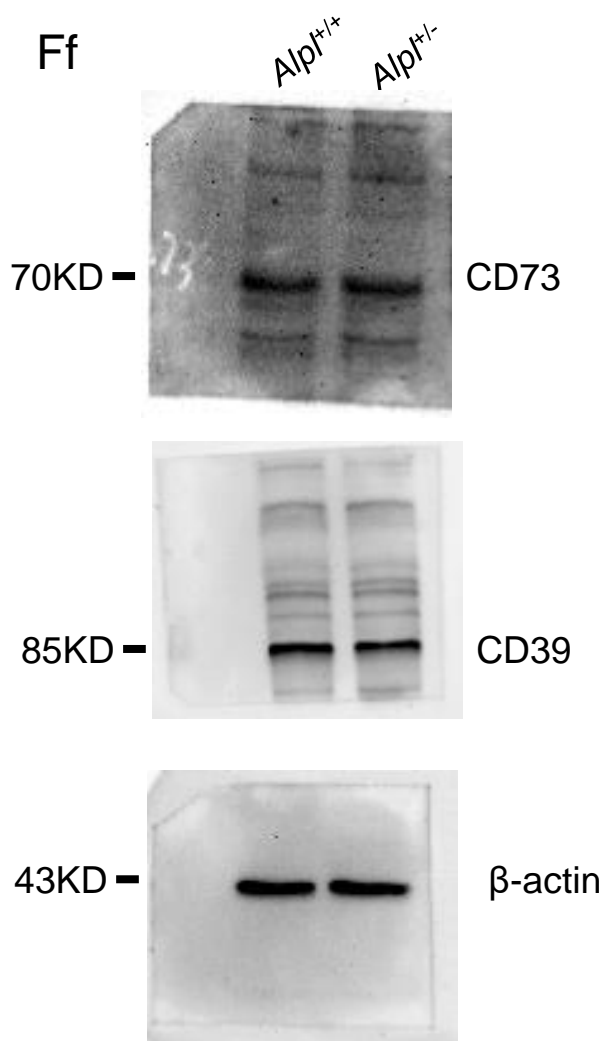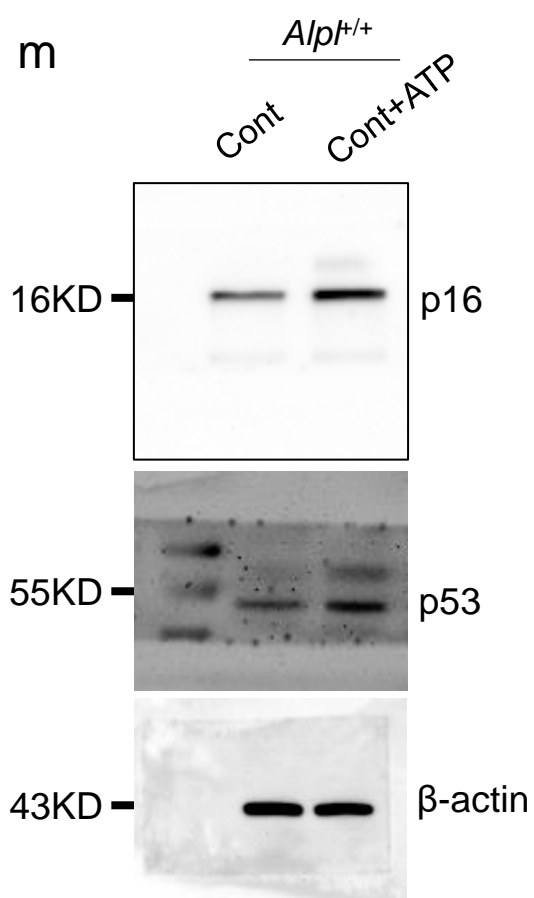

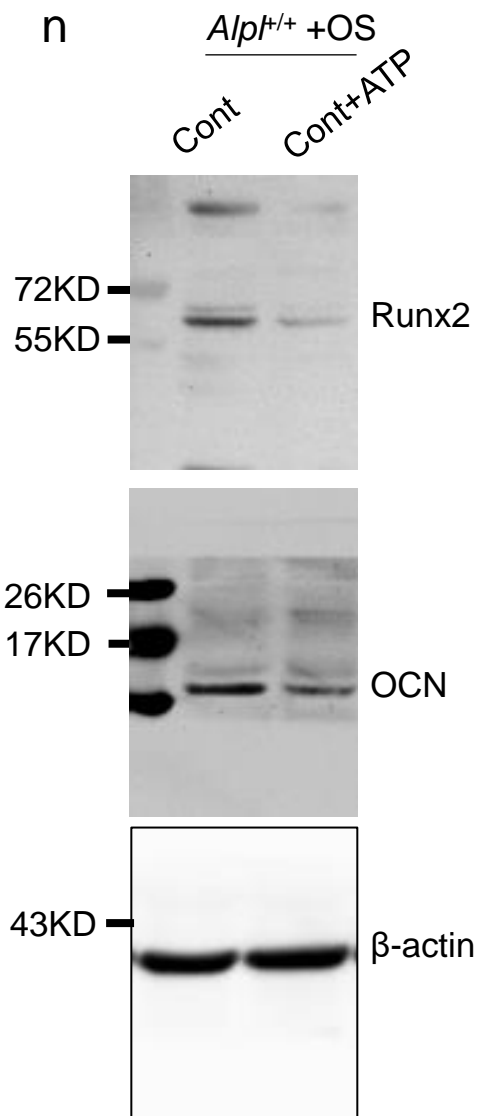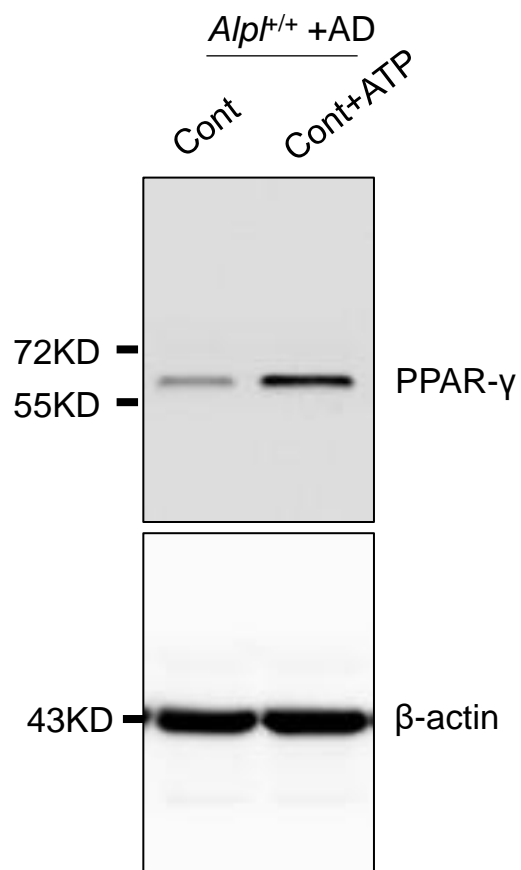

**Fig. 5**

**a**

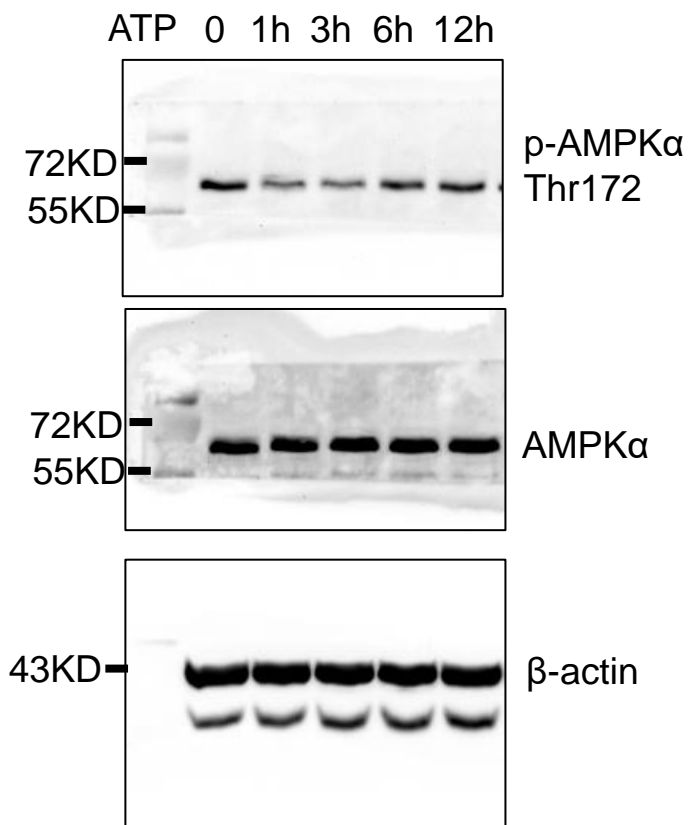

**b**

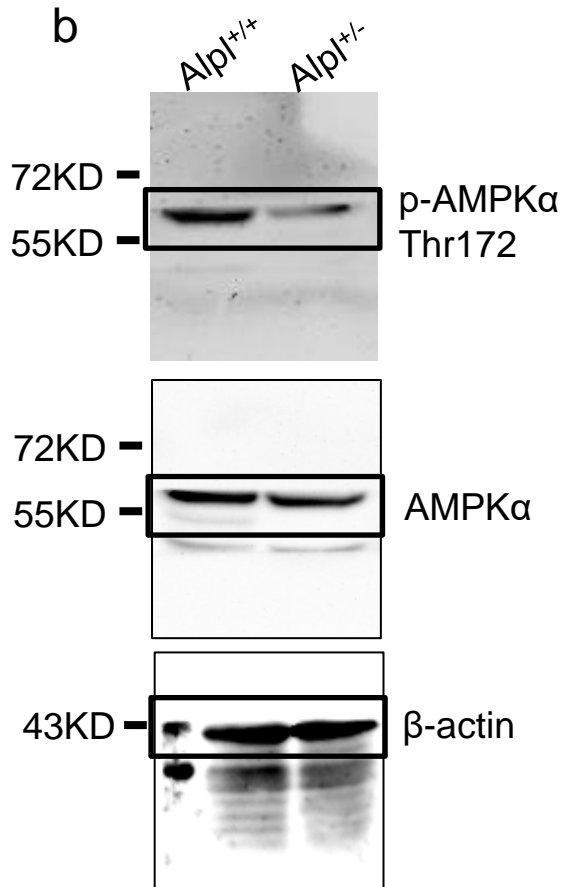

C

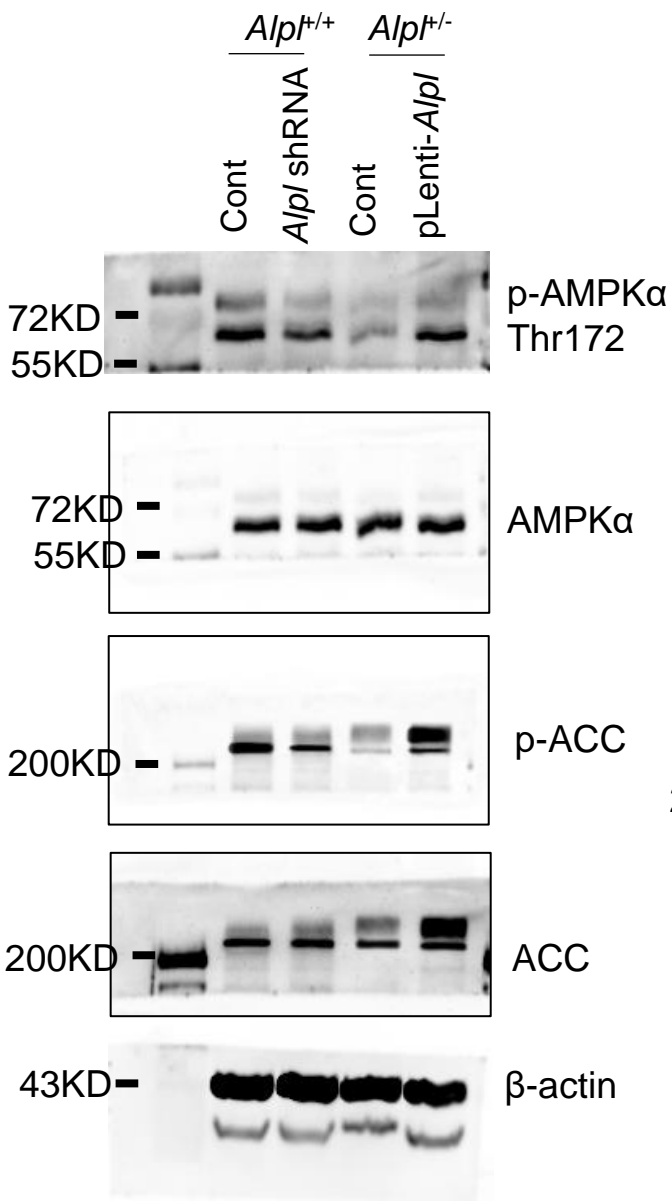

D

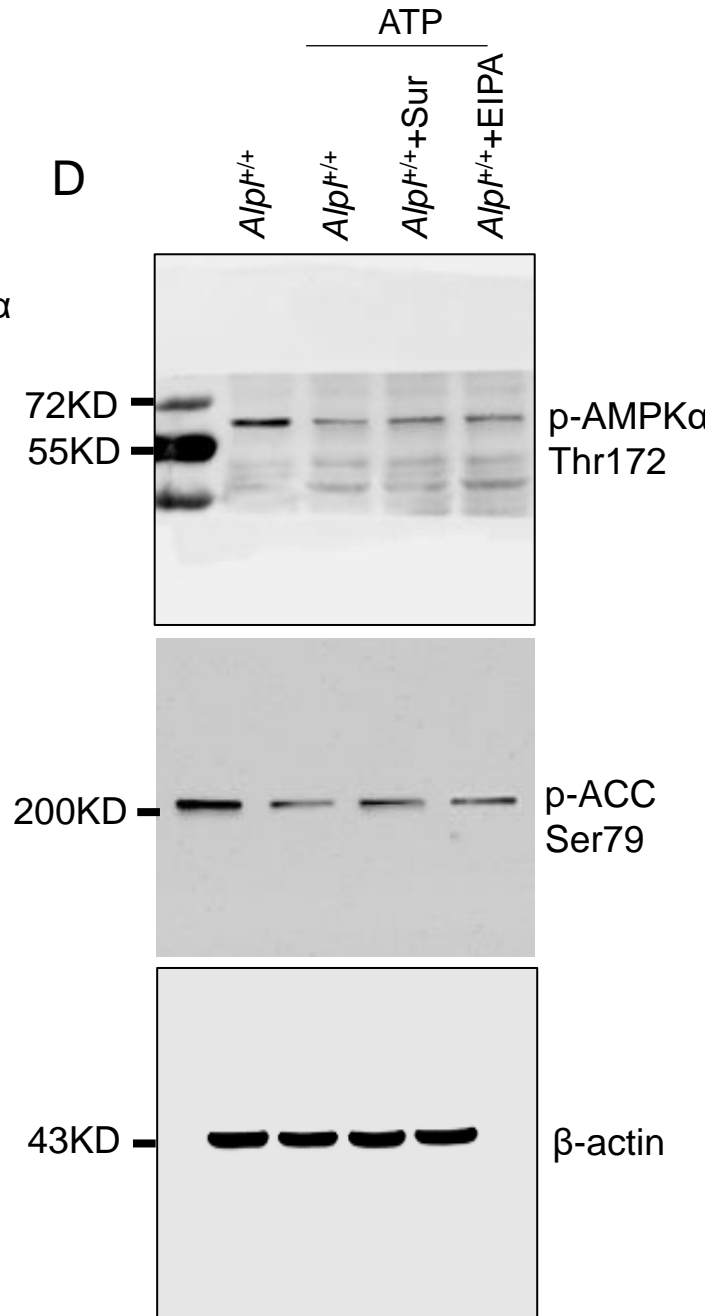

e

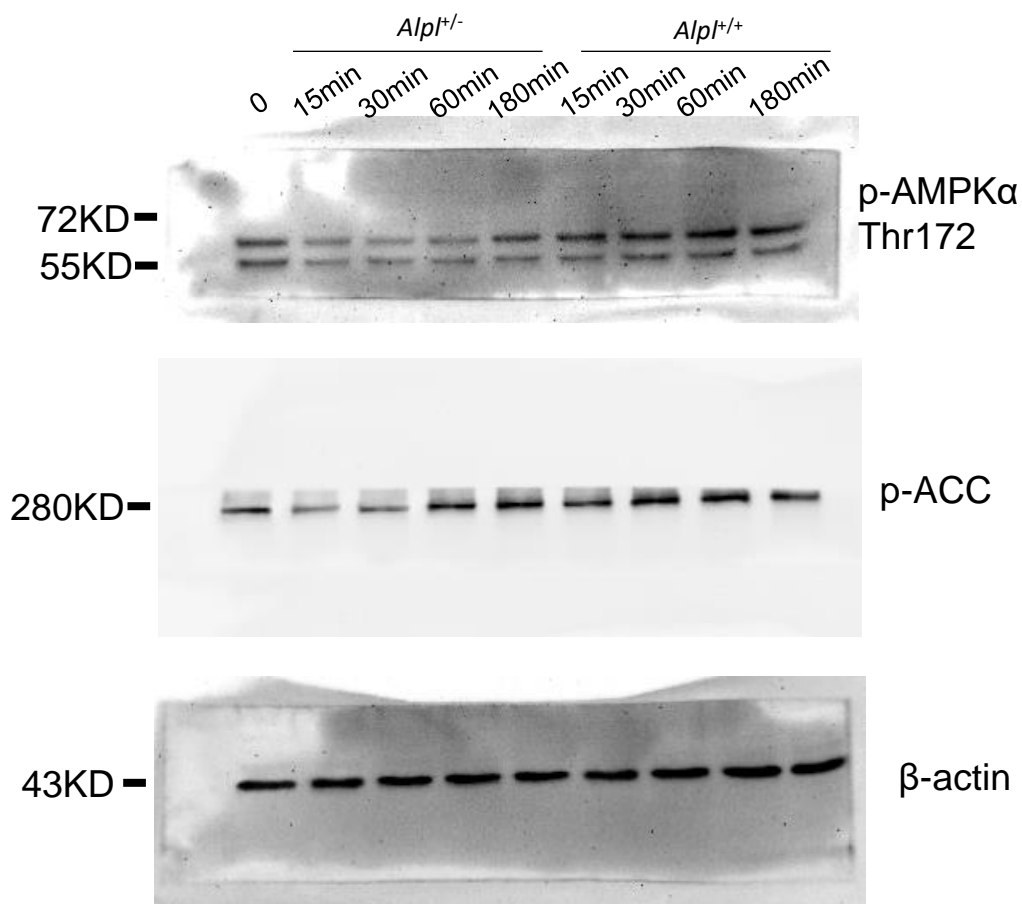

f

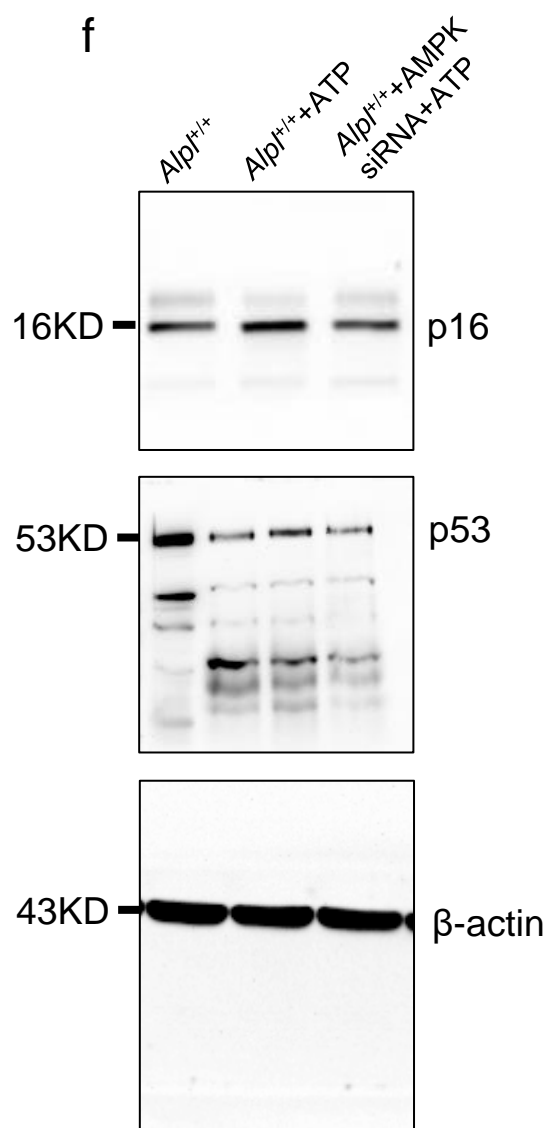

g

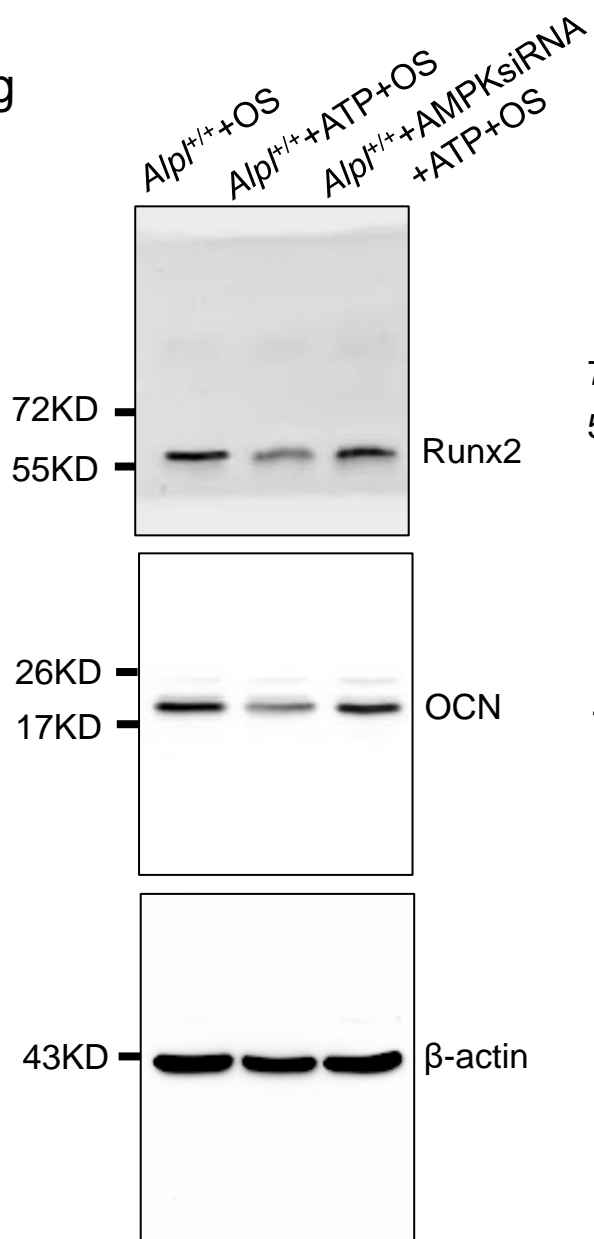

h

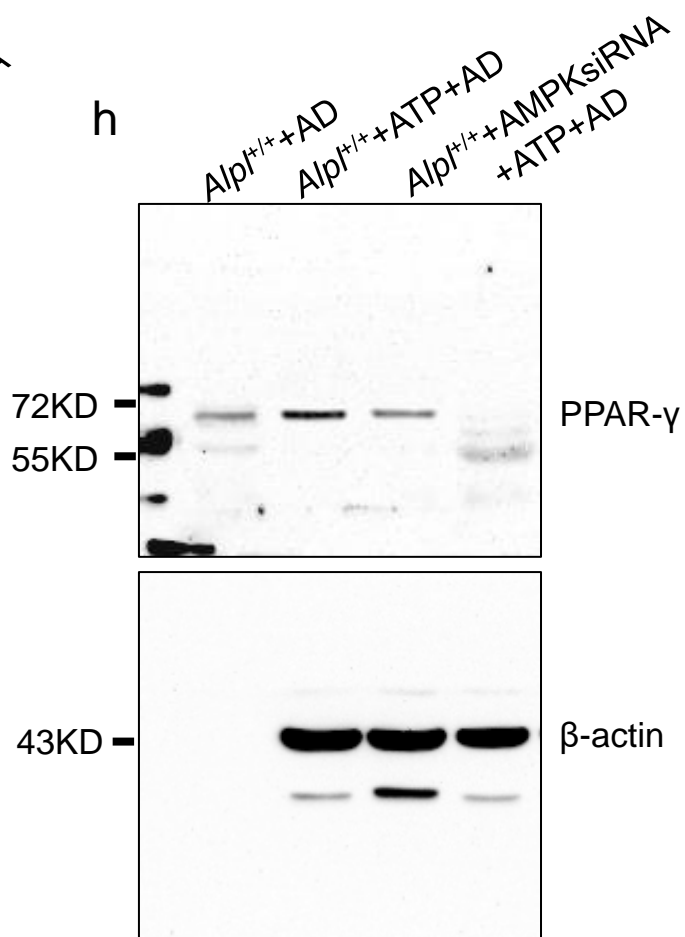

**Fig. 6**

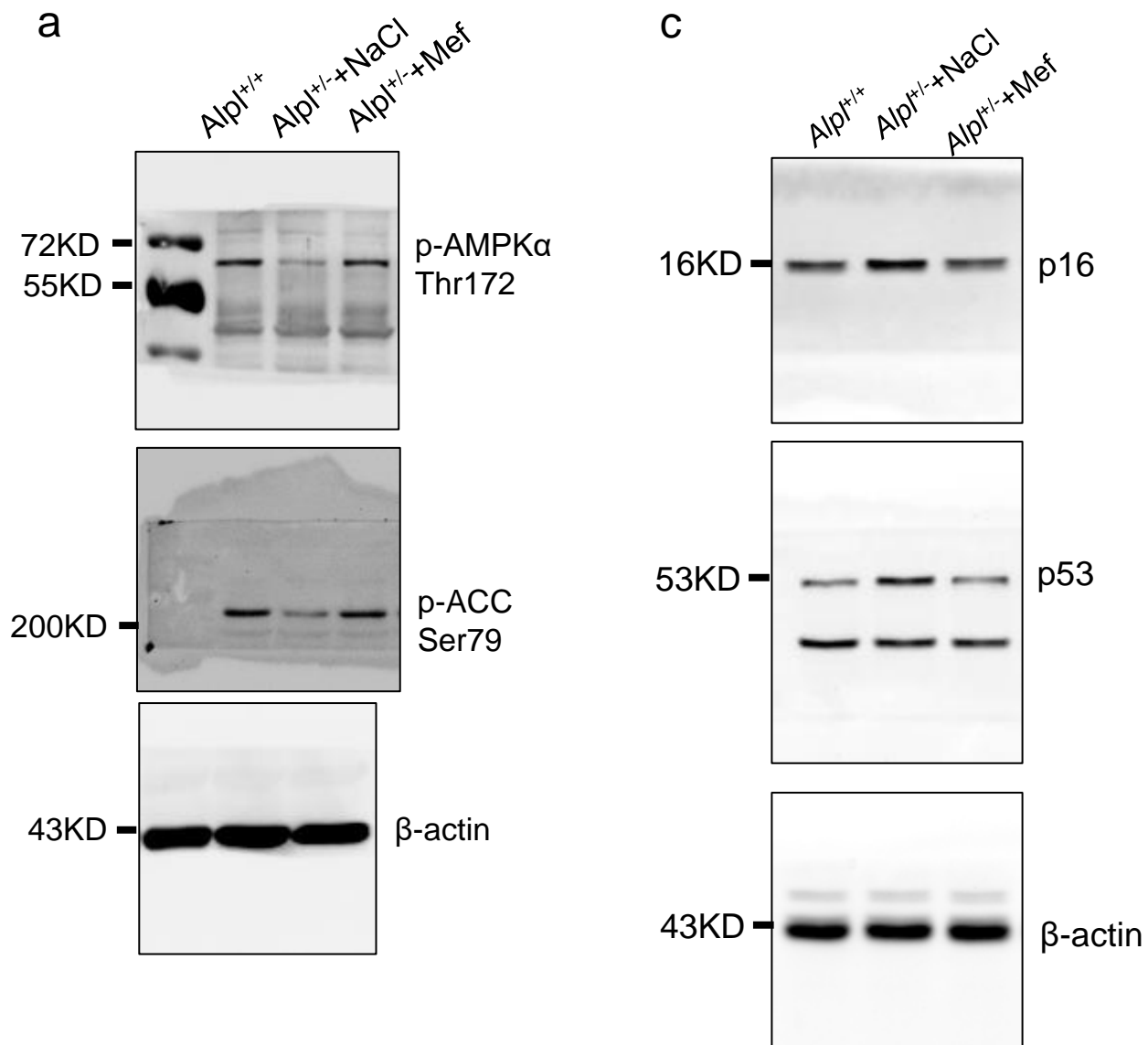

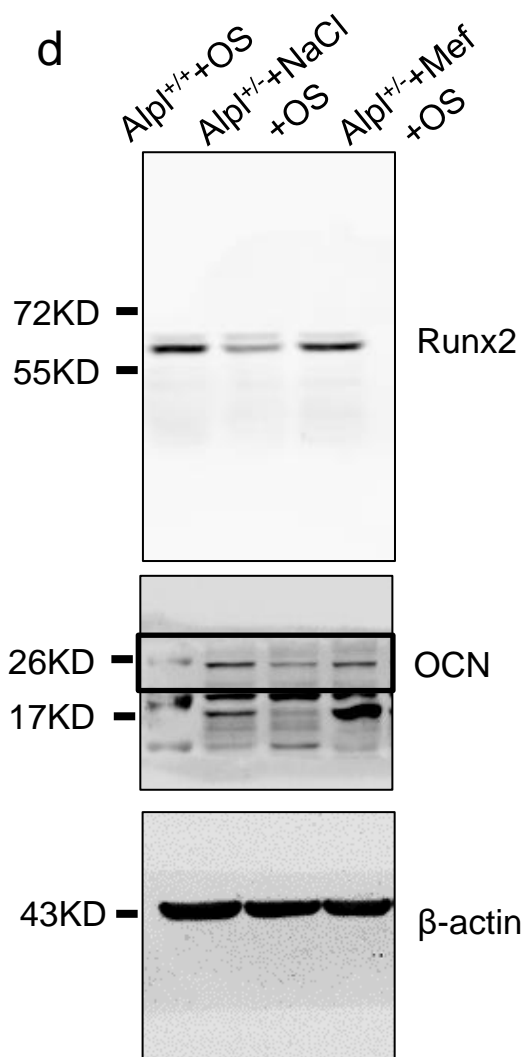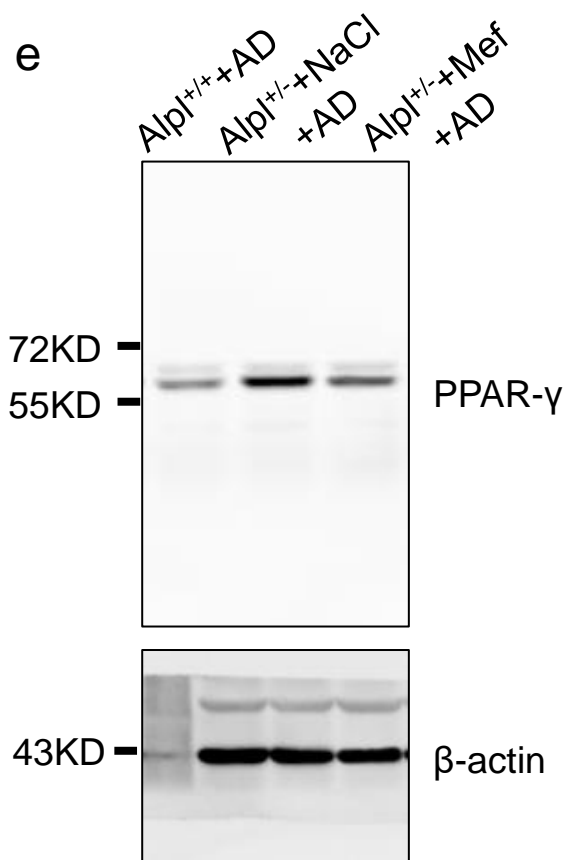

**Fig. 7**

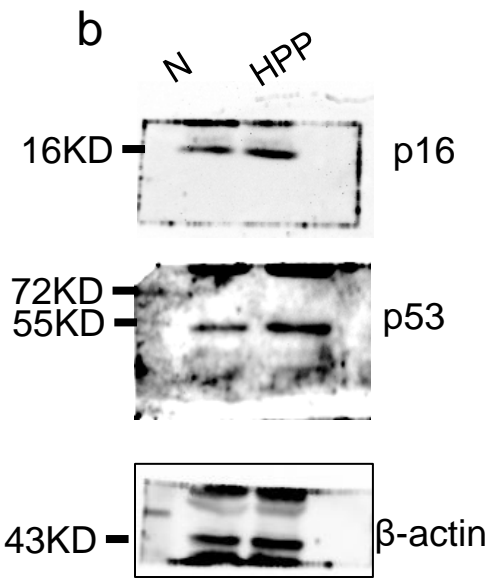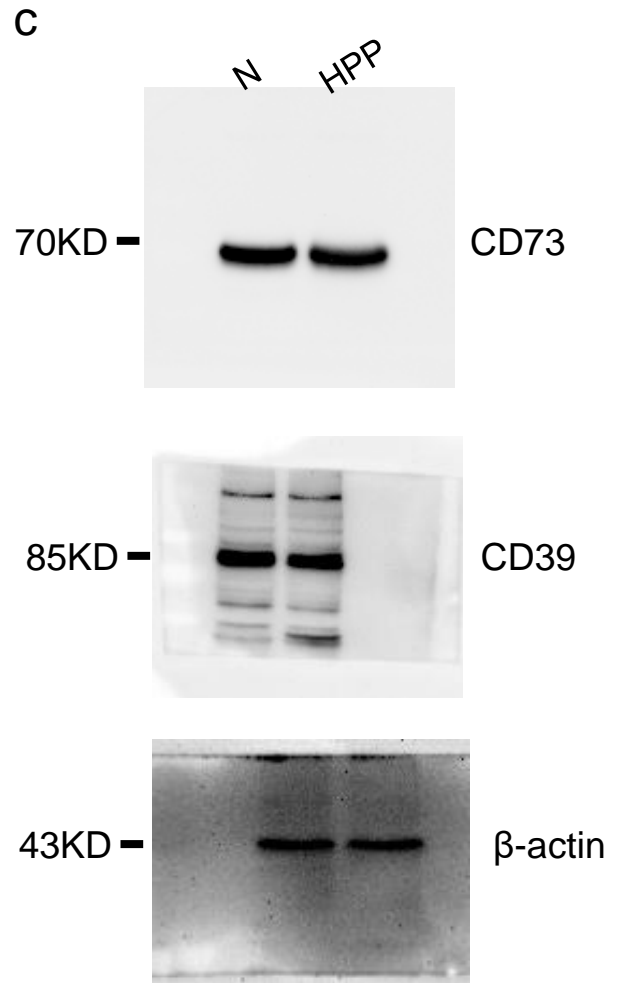

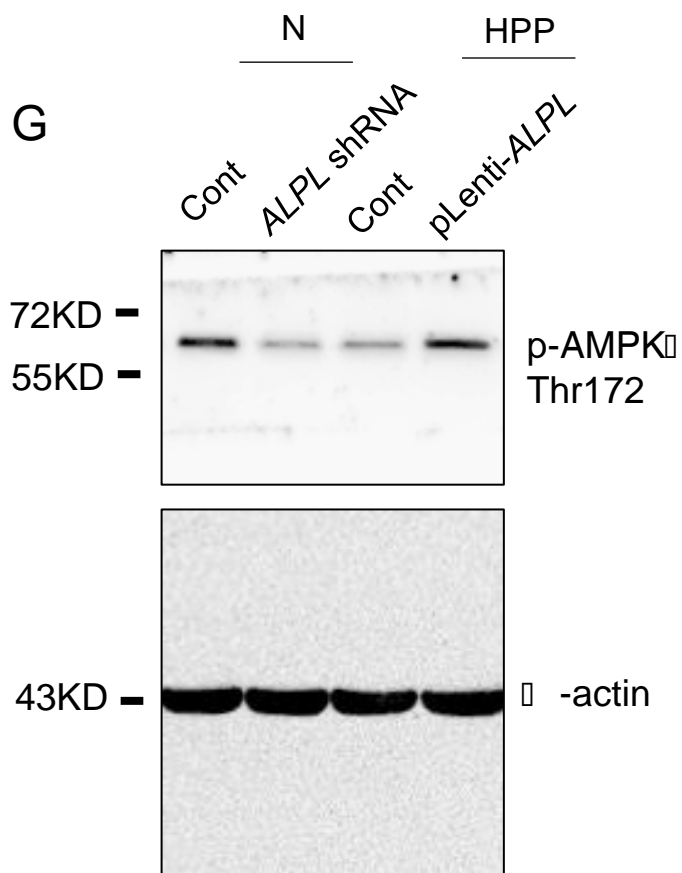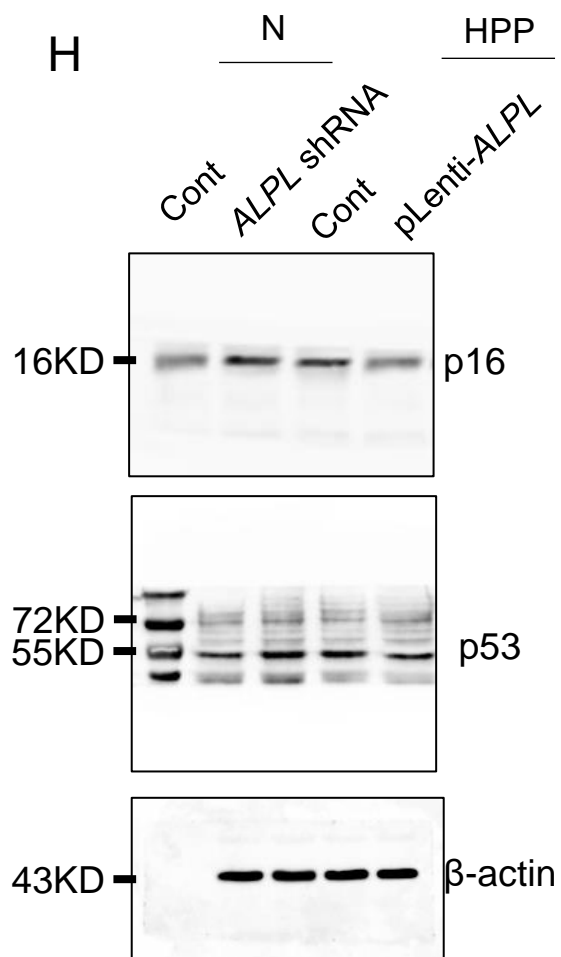

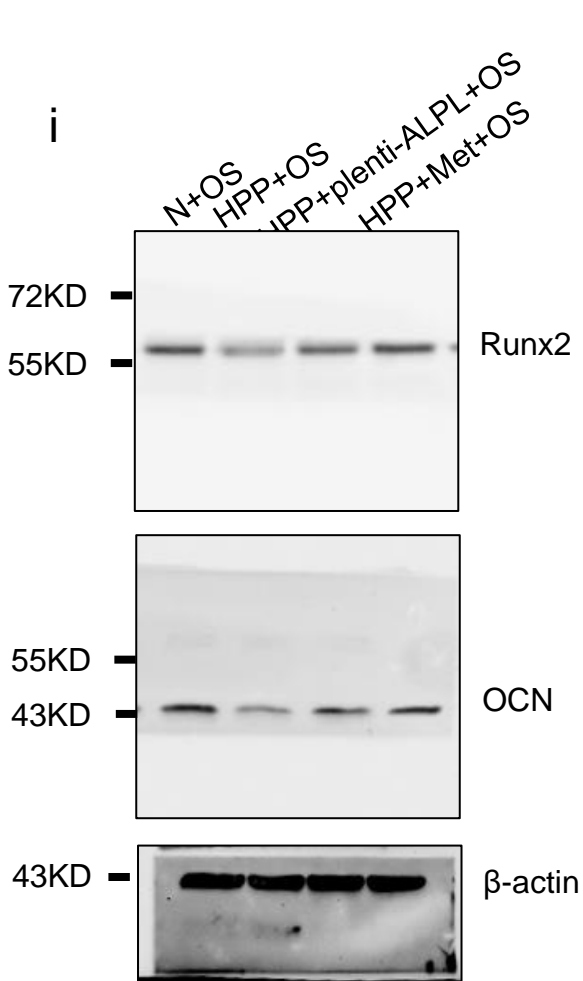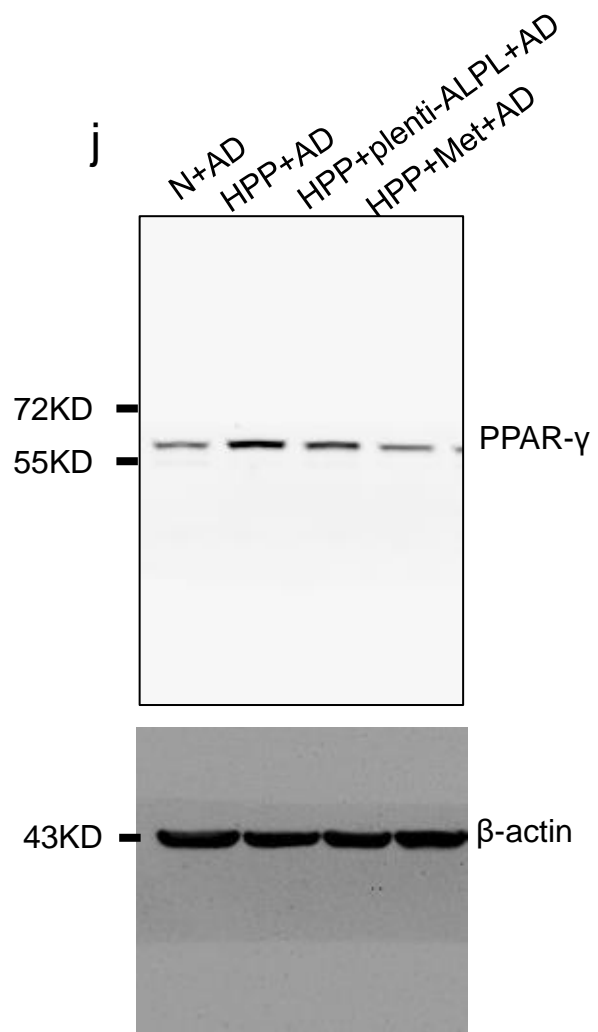

# Supplementary Fig. 3

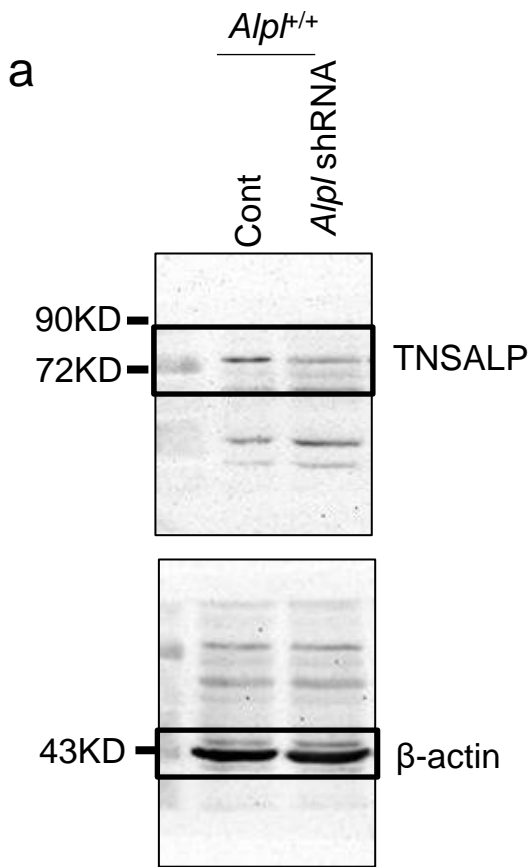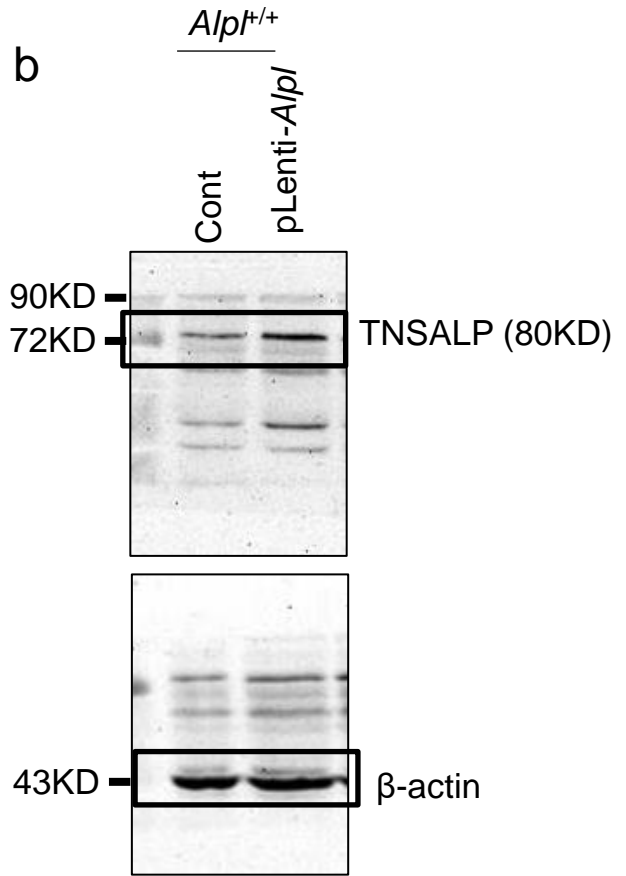

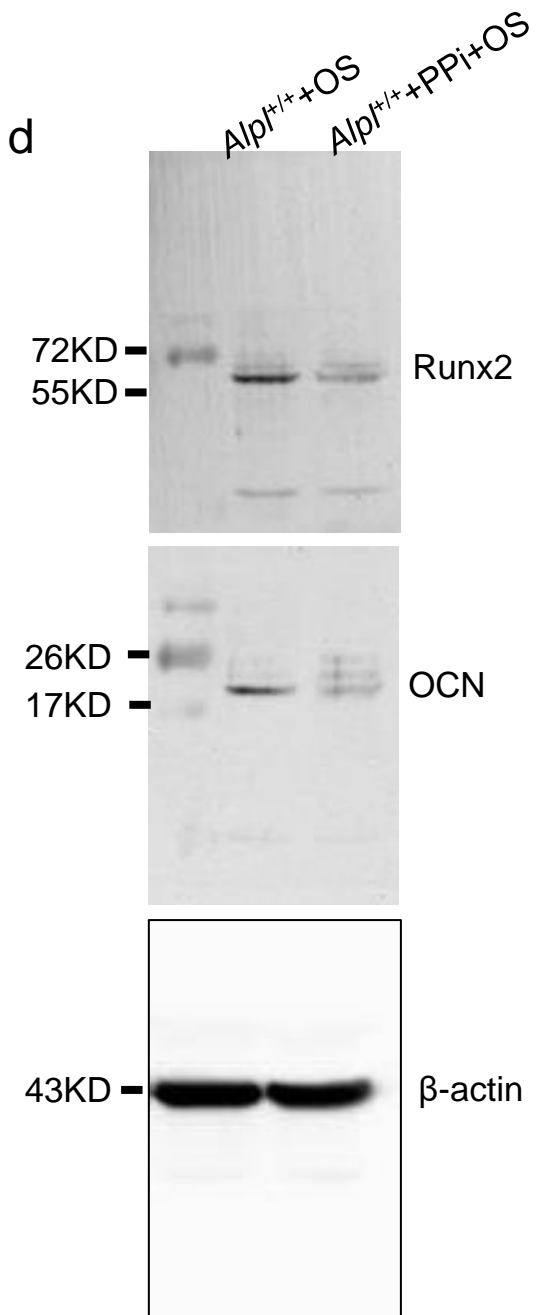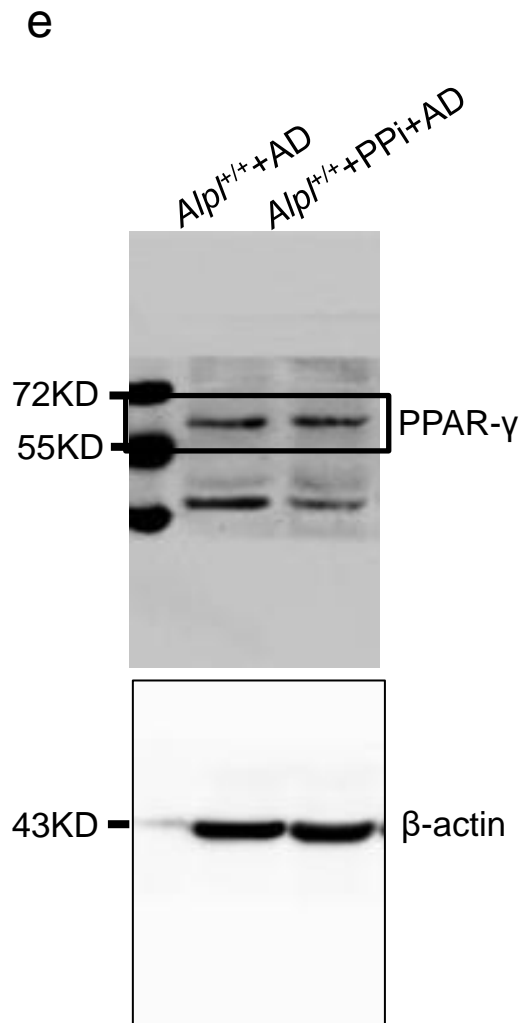

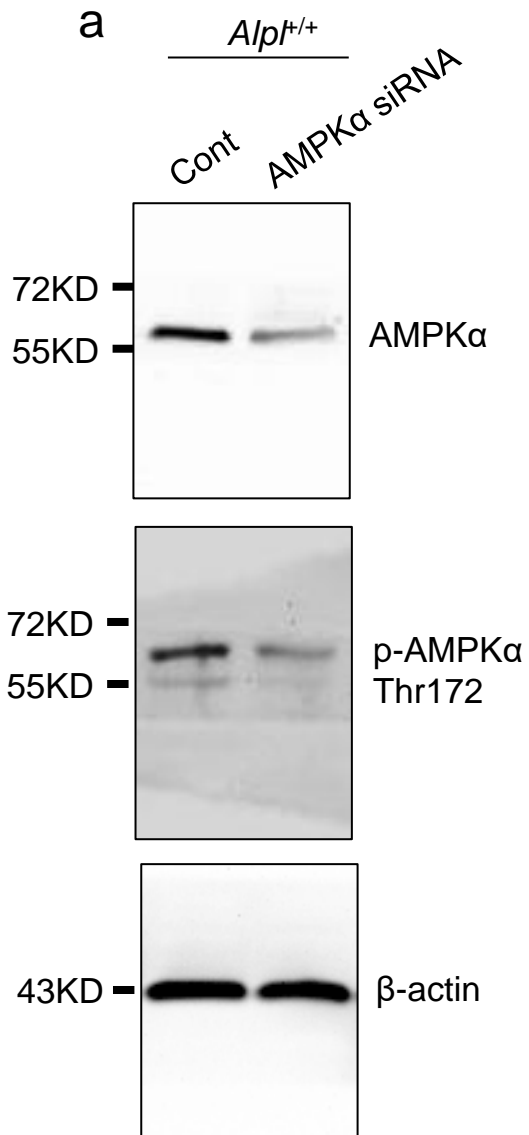

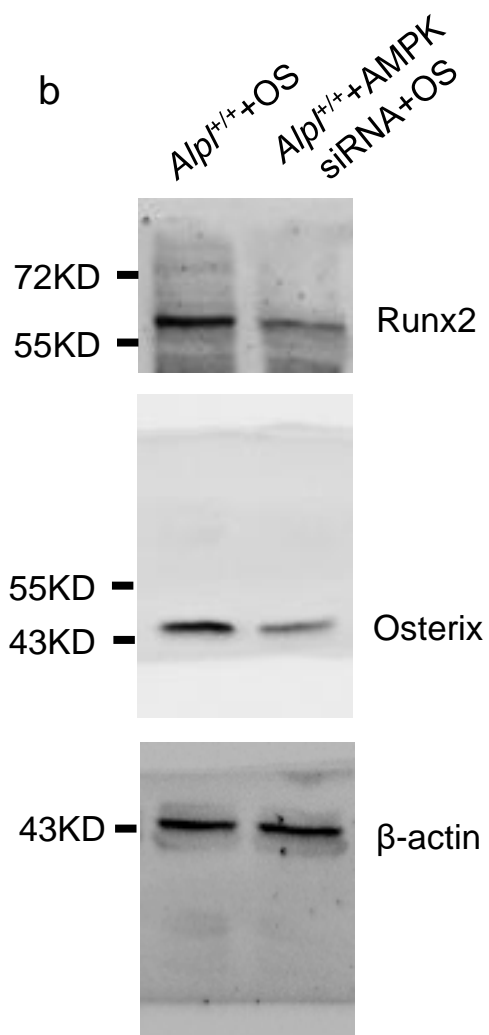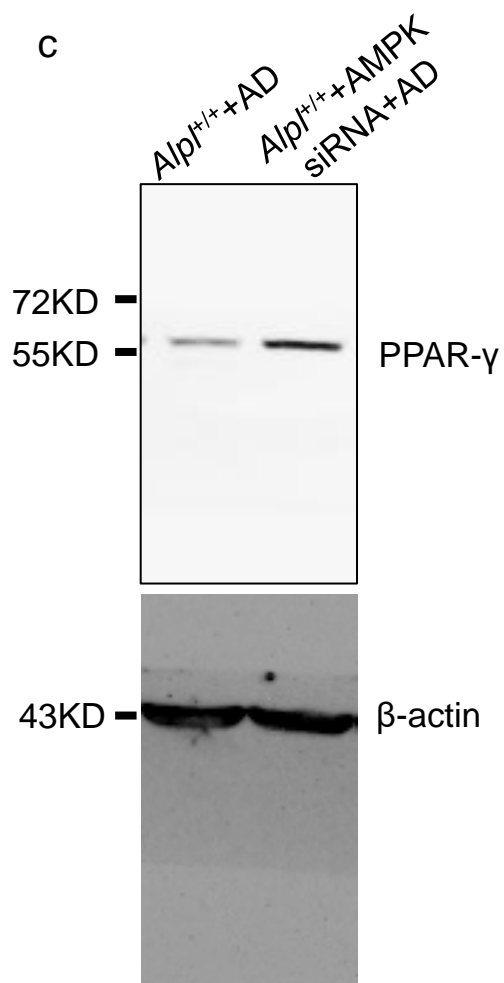

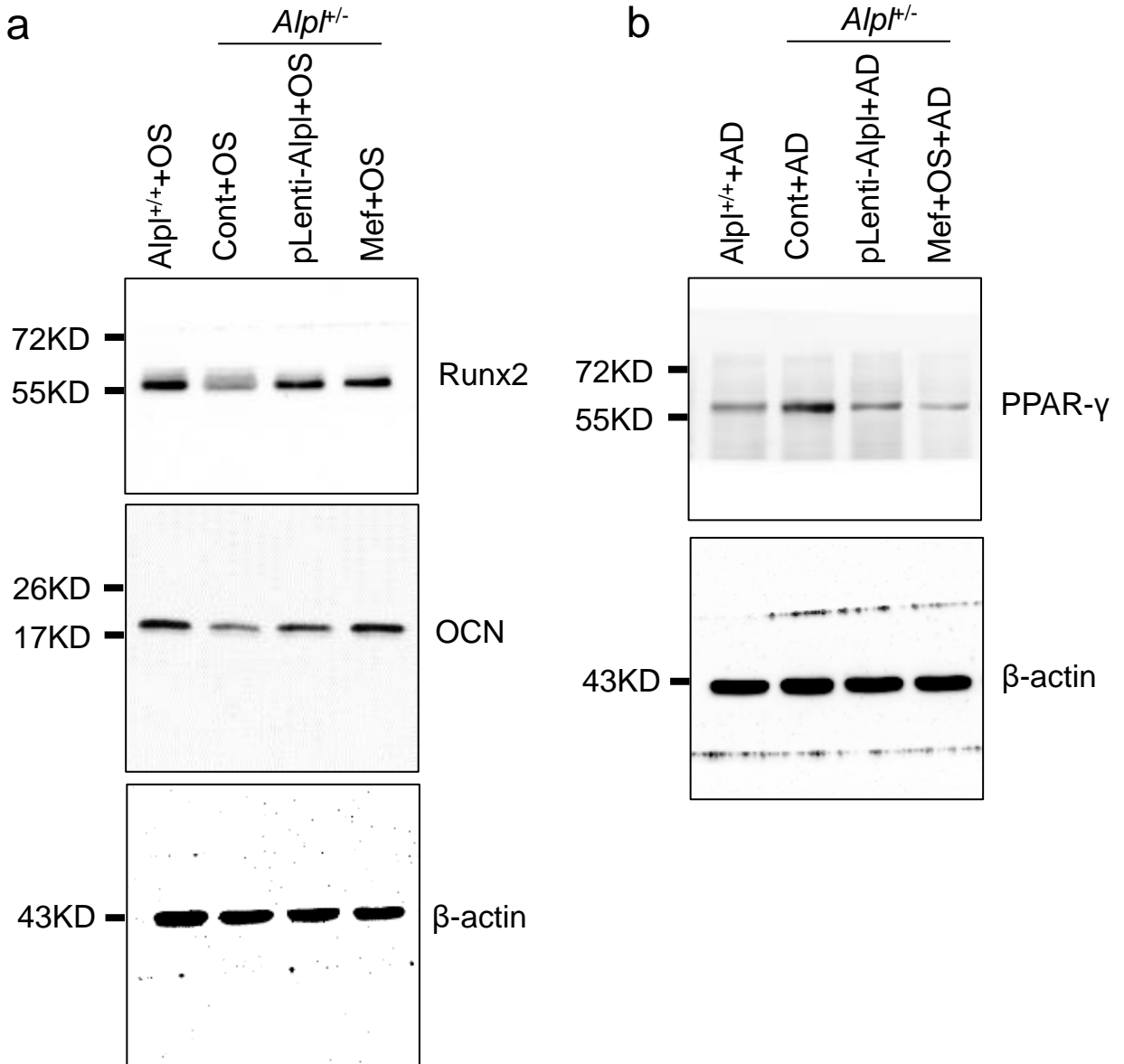

Supplement: Supplementary file 3 — Revised full uncutted gels [file 41413_2018_29_MOESM3_ESM.pdf]
