# Supplementary material for: Alpl prevents bone ageing sensitivity by specifically regulating senescence and differentiation in mesenchymal stem cells
Source: Bone Res. 2018 Sep 11;6:27. doi: 10.1038/s41413-018-0029-4 (PMC6131243; doi:10.1038/s41413-018-0029-4)
Supplement: Supplementary file 4 — Supplementary data [file 41413_2018_29_MOESM4_ESM.docx]

Supplementary Table 1. Identification of Mesenchymal Stem Cell Surface Markers by a Flow Cytometric Analysis.

| Mice | Sca-1 | CD73 | CD105 | CD90 | CD34 | CD45 | TNSALP |
| --- | --- | --- | --- | --- | --- | --- | --- |
| *Alpl*^+/+^ | 87.5% | 80.5% | 85.3% | 98.6% | 3.3% | 1.6% | 15.3% |
| *Alpl*^+/-^ | 79% | 78.1% | 80.5% | 98.8% | 2.9% | 1.8% | 7.8% |

Supplementary Table 2 Primers used to construct the *Alpl* lentiviral vector.

| Name | Sequence |
| --- | --- |
| *Alpl* shRNA | F-CCGGGCAGTATGAATTGAATCGGAACTCGAGTTCCGATTCAATTCATACTGCTTTTTG  R-AATTCAAAAAGCAGTATGAATTGAATCGGAACTCGAGTTCCGATTCAATTCATACTGC |
| pLenti  -*Alpl* | F-ACTGGATCCCAGCGAGGGACGAATCTCAGG  R-TATCTCGAGGGGGAGCTGGCTGTCCATTG |

Primers used to construct the *ALPL* lentiviral vector.

| Name | Sequence |
| --- | --- |
| ALPL shRNA1 | 5'-AATTCAAAAACATTCTCAAAGCCTCTTATTTCTCGAGAAATAAGAGGCTTTGAGAATG-3'  5'-CCGGCATTCTCAAAGCCTCTTATTTCTCGAGAAATAAGAGGCTTTGAGAATGTTTTTG-3' |
| ALPL shRNA2 | 5'-CCGGTTTGGCCAACAGGGTAGATTTCTCGAGAAATCTACCCTGTTGGCCAAATTTTT-3'  5'-AATTCAAAAATTTGGCCAACAGGGTAGATTTCTCGAGAAATCTACCCTGTTGGCCAAA-3' |
| pLenti  -ALPL | 5'-CTGGATCCGACCATGATTTCACCATTCTT-3'  5'-ATCTCGAGTGCCCTCAGAACAGGACGCT-3' |
